# Generation of Anti‐Mastitis Gene‐Edited Dairy Goats with Enhancing Lysozyme Expression by Inflammatory Regulatory Sequence using ISDra2‐TnpB System

**DOI:** 10.1002/advs.202404408

**Published:** 2024-08-05

**Authors:** Rui Feng, Jianglin Zhao, Qian Zhang, Zhenliang Zhu, Junyu Zhang, Chengyuan Liu, Xiaoman Zheng, Fan Wang, Jie Su, Xianghai Ma, Xiaoyu Mi, Lin Guo, Xiaoxue Yan, Yayi Liu, Huijia Li, Xu Chen, Yi Deng, Guoyan Wang, Yong Zhang, Xu Liu, Jun Liu

**Affiliations:** ^1^ Key Laboratory of Animal Biotechnology of the Ministry of Agriculture College of Veterinary Medicine Northwest Agriculture & Forestry University Yangling Shaanxi 712100 China

**Keywords:** gene‐editing breeding strategy, goats, ISDra2‐TnpB system, mastitis

## Abstract

Gene‐editing technology has become a transformative tool for the precise manipulation of biological genomes and holds great significance in the field of animal disease‐resistant breeding. Mastitis, a prevalent disease in animal husbandry, imposes a substantial economic burden on the global dairy industry. In this study, a regulatory sequence gene editing breeding strategy for the successful creation of a gene‐edited dairy (GED) goats with enhanced mastitis resistance using the ISDra2‐TnpB system and dairy goats as the model animal is proposed. This included the targeted integration of an innate inflammatory regulatory sequence (IRS) into the promoter region of the *lysozyme* (*LYZ*) gene. Upon *Escherichia Coli* (*E. coli*) mammary gland infection, GED goats exhibited increased LYZ expression, showing robust anti‐mastitis capabilities, mitigating PANoptosis activation, and alleviating blood‐milk‐barrier (BMB) damage. Notably, LYZ is highly expressed only in *E. coli* infection. This study marks the advent of anti‐mastitis gene‐edited animals with exogenous‐free gene expression and demonstrates the feasibility of the gene‐editing strategy proposed in this study. In addition, it provides a novel gene‐editing blueprint for developing disease‐resistant strains, focusing on disease specificity and biosafety while providing a research basis for the widespread application of the ISDra2‐TnpB system.

## Graphical abstract

This study presents a regulatory sequence gene‐editing breeding strategy and verifies its feasibility by creating mastitis‐resistant gene‐edited dairy goats (GED goats) using the ISDra2‐TnpB system. Upon *Escherichia Coli* mammary gland infection, GED goats exhibited significantly increased lysozyme expression, mitigation of PANoptosis activation, and alleviation of blood‐milk‐barrier damage, and reduced somatic cell count in milk, indicating robust anti‐mastitis capabilities.

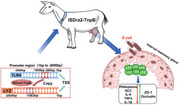

## 1 Introduction

Mastitis is a significant disease affecting livestock worldwide, causing significant economic losses and restricting industrial development.^[^ ^1^, ^2^ ^]^ The emergence of gene‐editing technology is expected to solve this problem. The ISDra2‐TnpB proteins have recently emerged as RNA‐guided nucleases capable of targeted genome editing in eukaryotic cells and represent a novel class of genome‐editing tools.^[^ ^3^ ^]^ Guided by reRNA (right element RNA, a long non‐coding RNA derived from the RE element in the ISDra2 transposon), ISDra2‐TnpB cleaves DNA near the 5ʹ end of the TTGAT transposition‐associated module (TAM), enabling cleavage of eukaryotic genomic DNA. Although studies have demonstrated the utility of the ISDra2‐TnpB system in implementing gene therapy in vivo, certain important questions remain unanswered, such as the feasibility of the ISDra2‐TnpB system for precise insertion of DNA fragments in the genome and the feasibility of generating gene‐edited animals.^[^ ^4^ ^]^ Currently, various transgenic mastitis‐resistant animals have been documented, including dairy goats with Toll‐Like Receptors 2 (TLR2) overexpression, dairy goats expressing the recombinant human *β‐defensin‐3* gene, dairy goats expressing the human *LYZ* gene, and dairy cows expressing the *lysostaphin* gene; however, it is difficult to apply in animal husbandry.^[^ ^2^, ^5^ ^]^ Recent reports indicate the imminent market entry of gene‐edited pigs resistant to blue ear disease, signifying consumer acceptance of gene‐edited animals lacking exogenous genetic material.^[^ ^6^ ^]^ Therefore, there is an urgent need to develop new strategies for mastitis‐resistant gene‐edited animals without exogenous genes to address the economic losses caused by mastitis and its impact on human health.

When pathogenic bacteria cause mastitis, it results in an intense inflammatory response and BMB damage within the mammary gland, during which a substantial influx of immune cells into the mammary gland exacerbates mastitis progression.^[^ ^7^ ^]^ The BMB's primary structure comprises tight junctions (TJ) between adjacent mammary epithelial cells that are essential for immune and physical protection.^[^ ^8^ ^]^ Therefore, effective mastitis management requires pathogen eradication and the preservation of BMB integrity and permeability to impede disease progression. According to the gene‐editing strategy proposed in this study, targeted editing of genes that are both bactericidal and anti‐inflammatory is required. The *LYZ* gene present in mammalian milk, is an ideal target gene and serves as a pivotal component of mammary gland immune defense.^[^ ^9^ ^]^ LYZ demonstrated potent antibacterial activity against gram‐positive and gram‐negative bacteria, as demonstrated by the ability of cattle milk containing the human *LYZ* gene to eliminate pathogenic bacteria in vitro efficiently.^[^ ^10^ ^]^ In addition to its antibacterial effects, LYZ exhibits anti‐inflammatory properties supported by studies demonstrating that supplementing LYZ can reduce the expression of pro‐inflammatory factors and alleviate inflammation.^[^ ^11^ ^]^ However, the anti‐inflammatory effects of LYZ in dairy goats have not been clarified, and further validation is needed. Recent studies have shown that the activation of PANoptosis perpetuates continuous inflammation in inflammatory diseases, underscoring its significance in disease progression.^[^ ^12^ ^]^ However, the detailed mechanism through which infectious inflammation lead to BMB damage remains unclear. Therefore, exploring the relationship between the occurrence of PANoptosis and mastitis can elucidates the pathogenesis of mastitis and is of great significance for the radical cure of mastitis and BMB damage.

In this study, we proposed a new gene‐editing strategy to combat inflammatory diseases caused by bacterial infections, which is the targeted integration of IRS into the promoter region of disease resistance genes and verified its feasibility. Using dairy goats as model animals, mastitis‐resistant GED goats were successfully generated using the ISDra2‐TnpB system, representing a marked improvement in biosafety compared with conventional transgenic approaches. During *E. coli* infection, GED goats exhibited increased LYZ in the mammary glands, improving their disease resistance. Additionally, our study confirmed the involvement of PANoptosis in BMB damage during mastitis pathogenesis and shed light on the potential regulatory mechanism of LYZ's anti‐inflammatory effect through regulating high mobility group box 1 (HMGB1) expression. Overall, the strategy focuses on disease‐specific, and biosafety, thus transcending the constraints of conventional breeding methods and the limitations associated with transgenic approaches. It holds significant importance for animal disease resistance breeding and provides a research basis for the wide application of the ISDra2‐TnpB system.

## 2 Results

### 2.1 Analysis of the Anti‐inflammatory Effect of Dairy Goat LYZ

The expression of different types of LYZ in the mammary glands of goats was analyzed using the Ruminant Genome.^[^ ^13^ ^]^ The results showed that *LYZ*, *LYZ C* (milk isozyme), *LYZ C* (intestinal isozyme), *LYZ C* (tracheal isozyme), *LYZ C‐1* and *LYZ like‐6* were expressed across various stages of mammary gland development, including 1, 3 and 9 months, early lactation, late lactation, and the dry period, but with wide variations in the expression levels (Figure S1a–f, Supporting Information). Notably, only LYZ exhibited a high homology with the amino acid sequence of human LYZ, reaching 83.78% (Figure S2, Supporting Information). Therefore, LYZ was selected for follow‐up studies.

Subsequently, we assessed the effect of LYZ recombinant protein on the viability of primary dairy goat mammary epithelial cells (GMEC) using the cell counting kit‐8 (CCK‐8) assay as a means of screening for the optimal treatment concentration. The results indicated that the cell viabilities of primary GMEC were exposed to varying concentrations of LYZ for 6, 12, and 24 h were not affected by the this concentration range tested (Figure S3a–c, Supporting Information). However, treatment with 10 nM LYZ for 12 and 24 h significantly alleviated Lipopolysaccharide (LPS)‐induced decline in primary GMEC activity (Figure S3d–f, Supporting Information). Therefore, the LYZ treatment concentration was selected as 10 nM for the subsequent experiments.

**Figure 1:**
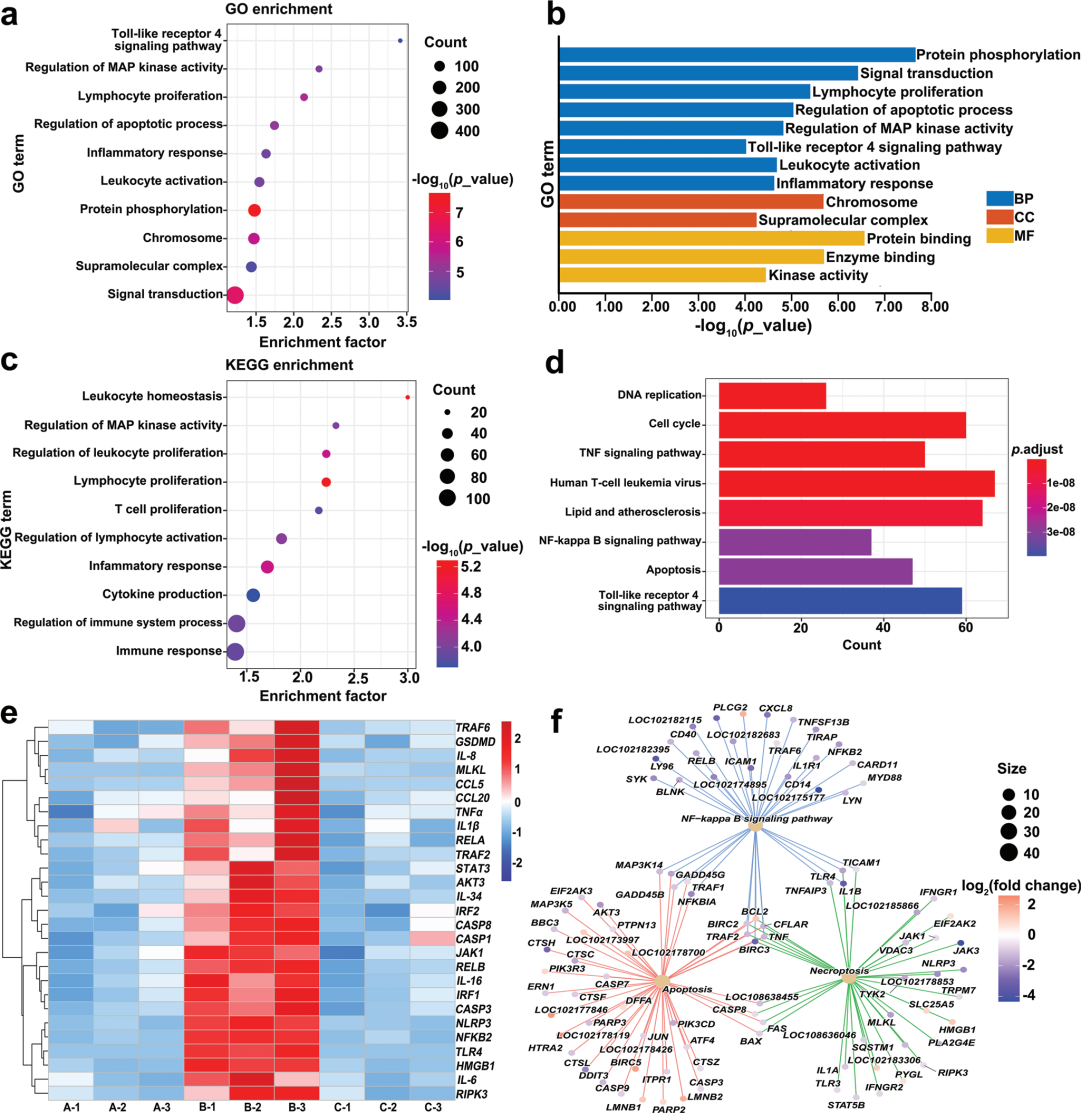
The anti‐inflammatory effects of dairy goat LYZ were analyzed by RNA‐seq. The cells were divided into 3 groups for treatment: group A (n = 3 per group) was the control group, group B (n = 3 per group) was treated with 5 µg mL^−1^ LPS for 12 h and group C (n = 3 per group) was treated with 5 µg mL^−1^ LPS for 12 h, and then 10 nM dairy goat LYZ recombinant protein was treated for 12 h. a) Bubble map obtained by GO analysis of RNA‐seq data from group B and C, with enrichment factors represented by horizontal coordinates. Bubble color and size correspond to the ‐log_10_(*p_*value). b) Bar graph obtained by GO analysis of RNA‐seq data from groups B and C, where the horizontal coordinate is the ‐log_10_(*p_*value). c) Bubble map obtained by KEGG analysis of RNA‐seq data from group B and C, where the horizontal coordinate is the enrichment factor. Bubble color and size correspond to the ‐log_10_(*p_*value). d) Bar graphs obtained by KEGG analysis of RNA‐seq data from groups B and C. Bar color correspond to the *p*.adjust. e) Heatmap analysis of RNA‐seq data from group A, B and C. f) Analysis of the apoptosis‐NF‐κB‐necrotosis interaction pathway was performed on the RNA‐seq data of group B and C. Bubble color and size correspond to the log_2_(fold change).

Primary GMEC treated with 5 µg mL^−1^ LPS for 12 h and 10 nM dairy goat LYZ recombinant protein for 12 h were used for RNA‐seq analysis to further verify the anti‐inflammatory effect of dairy goat LYZ. Gene Ontology (GO) enrichment analysis revealed the significant involvement of pathways such as the Toll‐Like Receptors 4 (TLR4) signaling pathway, mitogen‐activated protein kinase (MAPK) signaling pathway, apoptosis process, and inflammatory reaction (**Figure** **1**a,b). Similarly, Kyoto Encyclopedia of Genes and Genomes (KEGG) pathway analysis showed that significantly enriched pathways mainly involved the MAPK signaling pathway, inflammatory response, cytokine production, immune response, tumor necrosis factor (TNF) signaling pathway, nuclear factor‐kappa B (NF‐κB) signaling pathway, apoptosis signaling pathway and TLR4 signaling pathway (Figure 1c,d). These findings suggest that dairy goat LYZ regulates immune‐related processes and signaling pathways to alleviate primary GMEC inflammation. Heatmap analysis revealed that LPS‐induced inflammation in GMEC followed by the addition of dairy goat LYZ significantly reduced the expression of various pro‐inflammatory genes, including *TNF receptor associated factor 6* (*TRAF6*), *Interleukin‐8* (*IL‐8*), *Cysteine‐cysteine motif chemokine ligand 5* (*CCL5)*, *Cysteine‐cysteine motif chemokine ligand 20* (*CCL20*),*TNFα*, *Interleukin‐1β* (*IL‐1β*), *RELA Proto‐Oncogene, NF‐KB Subunit* (*RELA*), *TNF receptor associated factor 2* (*TRAF2*), *Signal transducer and activator of transcription 3* (*STAT3*), *Interleukin‐34* (*IL‐34*), *Interferon regulatory factor 2* (*IRF2*), *Janus kinase 1* (*JAK1*), *RELB Proto‐Oncogene, NF‐KB Subunit* (*RELB*), *Interleukin‐16* (*IL‐16*), *Interferon regulatory factor 1* (*IRF1*), *Nuclear factor kappa B subunit 2 (NFKB2*), *TLR4*, *HMGB1* and *Interleukin‐6* (*IL‐6*) (Figure 1e). Notably, genes associated with PANoptosis also exhibited downregulation, including *Gasdermin‐D* (*GSDMD*), *Mixed lineage kinase domain‐like protein* (*MLKL*), *NOD‐like receptor thermal protein domain associated protein 3* (*NLRP3*), *Cysteinyl aspartate specific proteinase 3* (*Caspase 3*), *Cysteinyl aspartate specific proteinase 8* (*Caspase 8*), *Cysteinyl aspartate specific proteinase 1* (*Caspase 1*) and *Receptor interacting protein kinase 3* (*RIPK3*) (Figure 1e). Furthermore, there is an association between the NF‐κB signaling pathway during the dairy goat LYZ‐mediated anti‐inflammatory process, apoptosis, and cell necrosis (Figure 1f). In summary, dairy goat LYZ has a favorable anti‐inflammatory effect by reducing the expression of pro‐inflammatory genes, thereby alleviating the inflammatory response.

### 2.2 The IRS Increased LYZ Expression in Gene‐edited GMEC and Alleviated Inflammation

**Figure 2:**
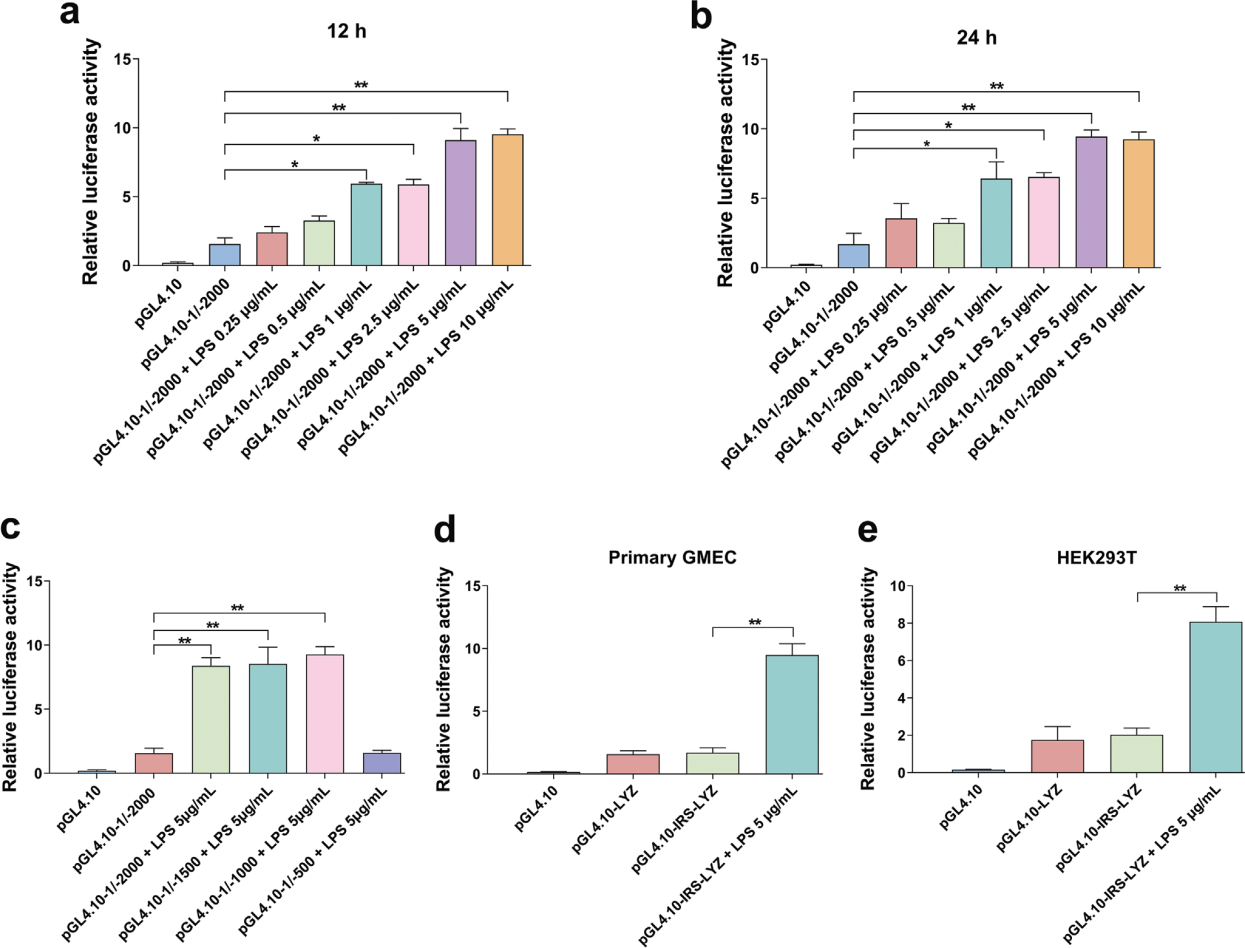
Screening of IRS. a,b) The pGL4.10‐1/−2000 vector was transfected into HEK293T cell and treated with LPS of different concentrations for 12 and 24 h to detect promoter activity. c) The pGL4.10‐1/−2000, pGL4.10‐1/−1500, pGL4.10‐1/−1000 and pGL4.10‐1/−500 vector were transfected into HEK293T cells, treated with 5 µg mL^−1^ LPS for 12 h to detect promoter activity. d,e) The pGL4.10‐IRS‐LYZ vector was transfected into primary GMEC and HEK293T cells, treated with 5 µg mL^−1^ LPS for 12 h to detect promoter activity. Values are expressed as mean ± SEM (n = 3 per group) by one‐way ANOVA. *: indicates significant difference (*P* < 0.05), **: indicates that the difference is highly significant (*P* < 0.01) and ns: indicates no significant difference (*P* > 0.05).

According to the regulatory sequence gene‐editing breeding strategy proposed in this study, a gene that is highly expressed only under inflammatory conditions and associated with the pathogenesis of mastitis needs to be screened for IRS. Moreover, heatmap results revealed that the expression of *TLR4* was significantly elevated in the GMEC inflammation model; therefore, *TLR4* gene was selected for IRS screening. Subsequently, the *TLR4* promoter region was obtained using PCR amplification, cloned into the pGL4.10 vector and transfected into HEK293T cells, which were treated with LPS at different concentrations for 12 h (**Figure** **2**a) and 24 h (Figure 2b). Analysis using a dual‐luciferase reporter system showed that the activity of *TLR4* promoter increased significantly with the treatment concentration and time. Additionally, truncation of the *TLR4* promoter region, cloned into the pGL4.10 vector, and transfected into HEK293T cells, followed by treatment with LPS for 12 h (Figure 2c), revealed a DNA sequence between −500 bp and −1000 bp from the transcription start site (TSS) that enhances *TLR4* promoter activity during LPS‐induced inflammation. This sequence also significantly enhanced *LYZ* promoter activity in primary GMEC (Figure 2d) and HEK293T cells (Figure 2e) and has been designated as IRS.

**Figure 3:**
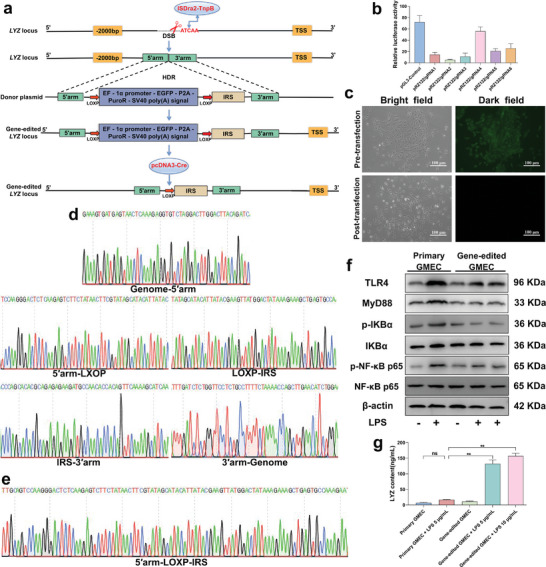
Analysis of anti‐inflammatory effects of gene‐edited GMEC. a) Gene editing schematic. b) Analysis of cleavage activity of six sgRNAs (n = 3 per group). c) Gene‐edited GMEC status before and after transfection with pCNDA3‐Cre vector. Scale bar: 100 µm. d,e) Sanger sequencing results of positive monoclones with green fluorescent screening markers and without screening markers. f) Primary GMEC and gene‐edited GMEC were treated with LPS for 12 h, and then the culture medium was replaced for 12 h to detect the protein expression level of TLR4, MyD88, p‐IKBα, IKBα, p‐NF‐κB‐p65 and NF‐κB‐p65 (n = 3 per group). g) Analysis of LYZ protein expression. Values are expressed as mean ± SEM (n = 3 per group) by one‐way ANOVA. *: indicates significant difference (*P* < 0.05), **: indicates that the difference is highly significant (*P* < 0.01) and ns: indicates no significant difference (*P* > 0.05).

The regulatory sequence gene‐editing breeding strategy used in this study is shown in the figure (**Figure** **3**a). First, single guide RNA (sgRNA) was designed to target the *LYZ* promoter region, and six single strand annealing (SSA) reporter plasmids, each containing the corresponding cleavage site, and six TnpB expression plasmids containing a 20‐nt guide sequence were constructed. Subsequently, these constructs were co‐transfected into HEK293T cells, and sgRNA activity was assessed using the SSA assay. Among the tested sgRNA, sgRNA4 exhibited the highest cleavage activity (Figure 3b). Therefore, sgRNA4 was selected for further experiments. However, in this study, the cleavage activity of CRISPR/Cas9 compared to that of ISDra2‐TnpB at the same cleavage site was unclear; therefore, sgRNAs of the corresponding CRISPR/Cas9 were designed at sgRNA1, sgRNA2, sgRNA3, sgRNA4, and sgRNA6 sequences to compare cleavage activity. The results showed that the cleavage activity of ISDra2‐TnpB was higher than that of CRISPR/Cas9 at the sgRNA4 position, which could be attributed to the easier and more efficient delivery of TnpB into the cell interior (Figure S4a–e, Supporting Information). Therefore, subsequent experiments were performed using the ISDra2‐TnpB system to obtain the gene‐edited cells.

We used the ISDra2‐TnpB system in combination with the Cre/LOXP system to obtain gene‐edited GMEC lacking foreign genes to verify whether IRS could enhance LYZ promoter activity and increase its expression in GMEC. The disappearance of green fluorescence confirmed the successful deletion of the foreign genes (Figure 3c). First, primary GMEC was electrotransfected with both target and donor vectors, enabling the integration of IRS into the *LYZ* promoter region via homology‐directed repair (HDR) and ensuring stable inheritance. Following purinomycin drug screening, drug‐resistant colonies harboring an accurately integrated IRS were identified using Sanger sequencing (Figure 3d; Figure S5a, Supporting Information). Cre recombinase vectors were electrotransfected into drug‐resistant colonies to delete foreign genes at two LOXP sites. Following Sanger sequencing analysis and the identification of positive monoclonal cell clusters devoid of foreign genes, these clusters were cultured (Figure 3e) (Figure S5b, Supporting Information). These cells exhibited no differences in viability compared to primary GEMC and were used for further experiments (Figure S6, Supporting Information).

Subsequently, an in vitro mastitis was established using LPS‐infected GMEC. Primary GMEC and gene‐edited GMEC were treated with different concentrations of LPS for 12 h, followed by culture in fresh medium for 12 h. Results indicated that LPS activation led to the stimulation of the TLR4/MyD88/NF‐κB signaling pathway, resulting in the production of inflammatory cytokines such as IL‐6, TNFα, and IL‐1β, thereby inducing an inflammatory response in primary GMEC (Figure 3f; Figure S7a–g and Figure S8, Supporting Information). However, gene‐edited GMEC showed increased LYZ expression (Figure 3g; Figure S7h, Supporting Information), inhibited the activation of TLR4/MyD88/NF‐κB signaling pathway, reduced the production of inflammatory factors such as IL‐6, TNFα, and IL‐1β, and alleviated the inflammatory response of gene‐edited GMEC under inflammatory stimulation (Figure 3f; Figure S7a–g and Figure S8, Supporting Information). In conclusion, IRS upregulates LYZ expression in gene‐edited GMEC under inflammatory conditions, thereby playing an anti‐inflammatory role.

### 2.3 Production of GED Goats using Somatic Cell Nuclear Transfer (SCNT)

**Figure 4:**
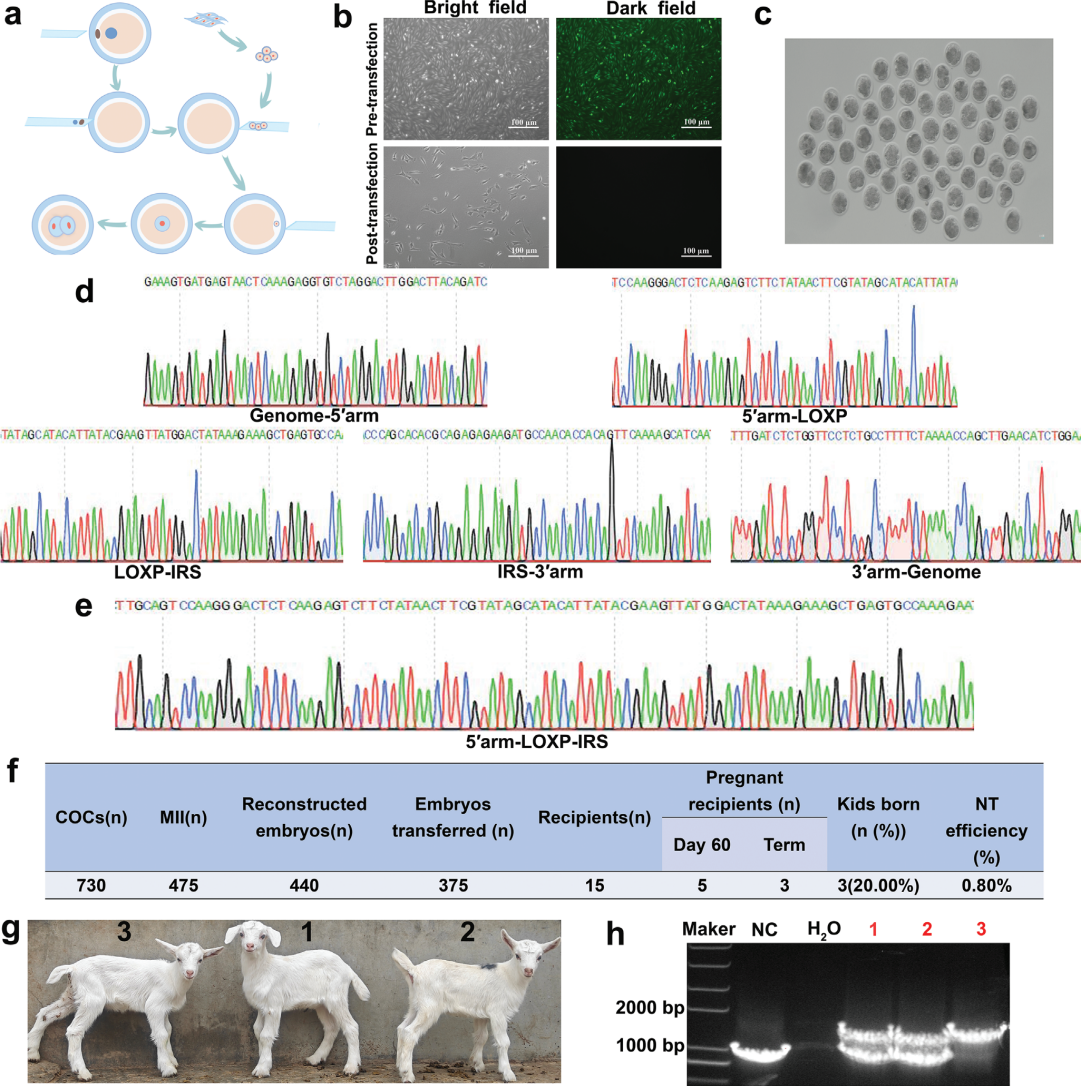
Production of LYZ GED goats by SCNT. a) Schematic diagram of SCNT. b) Gene‐edited GFFs status before and after transfection with pCNDA3‐Cre vector. Scale bar: 100 µm. c) Developmental status of embryos after SCNT. Scale bar: 100 µm. d,e) Sanger sequencing results of positive monoclones with green fluorescent screening markers and without screening markers. f) Production data of GED goats. g) Three GED goats. h) Identification of GED goats.

We determined that IRS could increase LYZ expression in gene‐edited GMEC and attenuate the inflammatory response; however, further validation is needed to determine whether IRS can increase LYZ expression in individuals and thereby reduce the severity of mastitis. Therefore, we screened goat fetal fibroblasts (GFFs) for SCNT to produce GED goats using the regulatory sequence gene editing breeding strategy proposed in this study (Figure 3a and **Figure** **4**a).

Positive monoclonal cells devoid of foreign genes were obtained using the ISDra2‐TnpB system along with the Cre/LOXP system. The disappearance of green fluorescence confirmed the successful deletion of the foreign genes (Figure 4b). Electrotransfection of the targeted and donor vector into GFFs, and facilitated the integration of IRS into the *LYZ* promoter region of GFFs, ensuring stable inheritance through HDR. After puromycin screening, 13 drug‐resistant colonies were subjected to 5ʹ‐junction PCR identification, resulting in the identification of 9 drug‐resistant colonies with accurate genomic integration (Figure S5a and Figure S9a, Supporting Information). Drug‐resistant colonies identified through 5ʹ‐junction PCR were further analyzed using 3ʹ‐junction PCR to ensure the accuracy of our findings, resulting in the selection of eight drug‐resistant colonies (Figure S5a and Figure S9b, Supporting Information). Sanger sequencing of the 3000 and 2200 bp fragments obtained from the 5ʹ‐junction PCR and 3ʹ‐junction PCR, confirmed the precise insertion site (Figure 4d). Subsequently, Cre recombinase was used to delete foreign genes in drug‐resistant colonies correctly identified using two rounds of junction PCR. Finally, accurately positive monoclonal cell clusters, which could be used for subsequent SCNT, were identified by analyzing PCR amplification products and Sanger sequencing (Figure 4e; Figure S5b, Supporting Information).

The obtained positive monoclonal cell clusters were cultured in a serum‐free medium for 24 h before SCNT (Figure 4a). A total of 440 reconstructed embryos were generated, among which 375 well‐developed cloned embryos were transferred to 15 healthy recipient ewes (Figure 4c). Finally, three GED goats were obtained, with a birth rate of 20% (Figure 4f,g). Subsequently, PCR amplification and analysis of the blood genomes of the three GED goats revealed that IRS localized to the *LYZ* promoter region (Figure 4h). Subsequently, we cloned seven main sgRNA4 potential off‐target sites that were predicted based on sequence similarity to the target sequence from all GED goat genomes. We did not detect any typical indels at any of the analyzed off‐target sites (Figure S10, Supporting Information). These results demonstrated the precise integration of the IRS at the *LYZ* promoter region without off‐target modifications being detected. Therefore, GED goats with IRS‐targeted integrations into the *LYZ* promoter region were successfully obtained for the subsequent analysis of mastitis resistance.

### 2.4 GED Goats with High LYZ Expression Reduce the Severity of Mastitis and Alleviate BMB Damage

Before establishing the mastitis model, mammary gland tissue samples surgically obtained from GED goats and GMEC were isolated for subsequent experiments (Figure S11, Supporting Information). We screened various concentrations of *E. coli* for their ability to induce mastitis in wild type dairy goat (WTD goats) to determine the optimal concentration of *E. coli* for inducing mastitis in GED goats. Mastitis onset was assessed based on somatic cell counts (SCC) index and expression levels of pro‐inflammatory factors. Finally, 6 × 10^6^ CFU mL^−1^ *E. coli* was selected for use in the GED goat mastitis model (Figure S12, Supporting Information).

**Figure 5:**
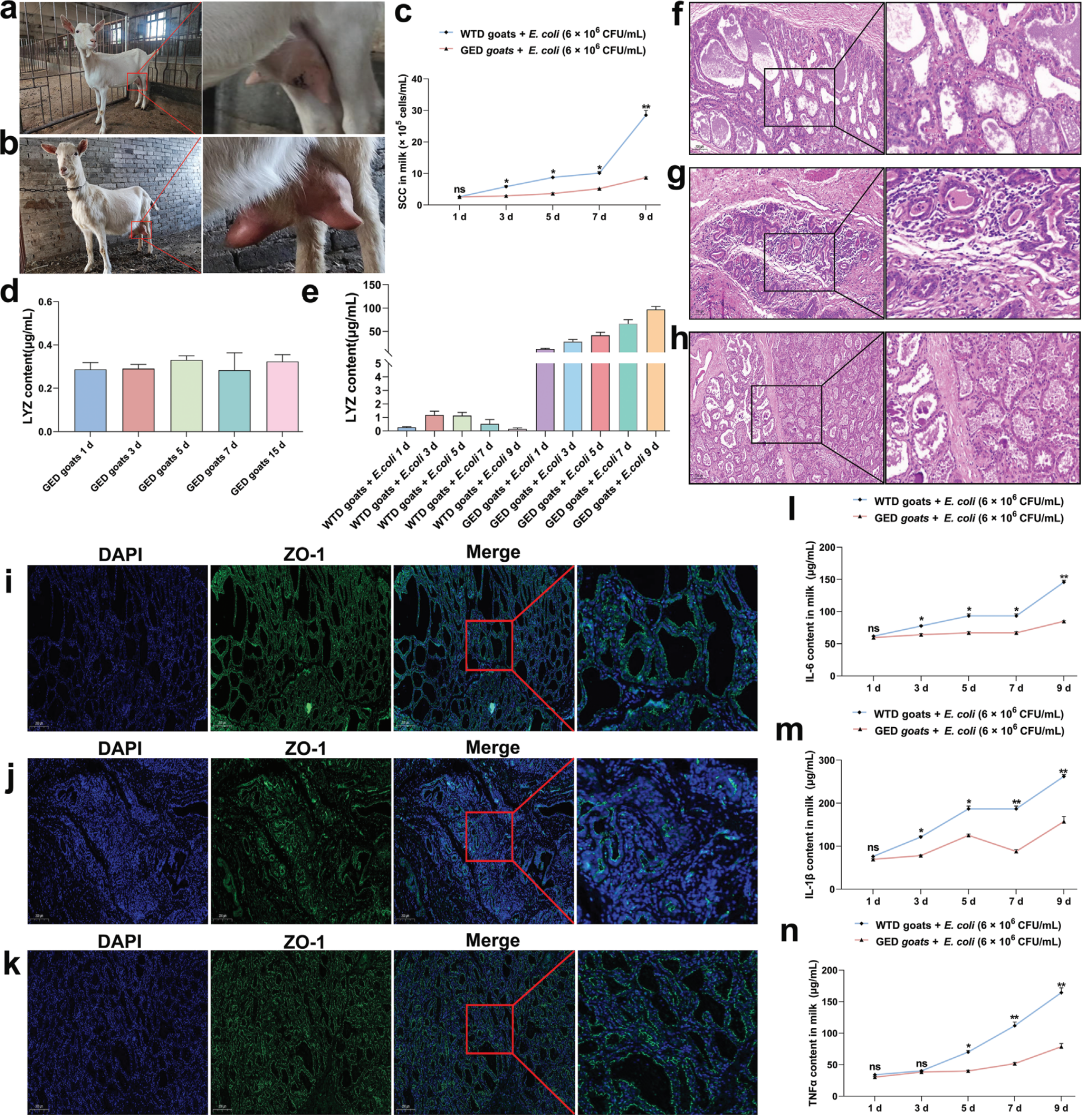
GED goats with high LYZ expression reduce the severity of mastitis and alleviate BMB damage. a,b) The status of GED goats and WTD goats after infection with *E.coli* for 7 d. c) SCC in milk during *E. coli* infection (n = 3 per group). d) Expression level of LYZ protein in GED goats milk 15 d before infection (n = 3 per group). e) Expression level of LYZ protein in WTD goats and GED goats milk after infection (n = 3 per group). f,g) HE staining of mammary gland tissue of WTD goats during lactation after 7 d of sterile PBS and *E.coli* injection, respectively. Scale bar: 100 µm. h) HE staining of mammary gland tissue of GED goats during lactation after 7 d of *E.coli* injection. Scale bar: 100 µm. i,j) ZO‐1staining of mammary tissue during lactation of normal WTD goats during lactation after 7 d of sterile PBS and *E.coli* injection, respectively. Scale bar: 200 µm. k) ZO‐1 staining of mammary tissue during lactation of GED goats during lactation after 7 d of *E.coli* injection. Scale bar: 200 µm. l–n) Analysis of content of IL‐6, TNFα, and IL‐1β in milk (n = 3 per group). Values are expressed as mean ± SEM (n = 3 per group) by one‐way ANOVA. *: indicates significant difference (*P* < 0.05), **: indicates that the difference is highly significant (*P* < 0.01) and ns: indicates no significant difference (*P* > 0.05).

An experiment using bacterial infection was conducted to determine the ability of GED goats to resist mastitis. Observations revealed that GED goats showed no abnormalities, displaying only slight redness and swelling in the teats (**Figure** **5**a), whereas the mammary glands of WTD goats exhibited redness, swelling, and became hard (Figure 5b). The SCC index of the milk from WTD goats was significantly higher than that of GED goats (Figure 5c). There was no significant change in the concentration of LYZ in GED goats prior to *E. coli* infection (Figure 5d); however, there was a significant increase in LYZ concentration following infection (Figure 5e). Hematoxylin‐eosin staining (HE) staining and immunofluorescence (IF) analysis revealed distinct differences between WTD and GED goats. WTD goats without *E. coli* infection exhibited intact BMB, absence of immune cell aggregation, and normal distribution of Zonula occludens‐1 (ZO‐1) protein in the mammary gland tissue (Figure 5f,i). Conversely, WTD goats infected with *E. coli* for 7 d showed a nearly complete loss of intact BMB in the mammary gland, extensive immune cell infiltration, and dispersed or absent ZO‐1 protein, indicative of the damage of BMB in the mammary gland (Figure 5g,j). In contrast, GED goats infected with *E. coli* for 7 d displayed intact BMB, minimal clustering of immune cells, and relatively normal ZO‐1 protein distribution in the mammary tissue (Figure 5h,k). Additionally, the expression of IL‐6, TNFα, and IL‐1β, was significantly lower in the milk of GED goats compared to that in WTD goats (Figure 5l–n). These findings suggested that IRS increases LYZ expression in the mammary glands of GED goats during mastitis and that the high expression of LYZ in GED goats exhibited resistance to mastitis induced by *E. coli* infection, reduced the expression of SCC and pro‐inflammatory factors in milk, and demonstrated a mitigating effect on BMB damage.

### 2.5 Gene‐edited GMEC Alleviates Inflammation and TJ Damage by High Expression of LYZ

**Figure 6:**
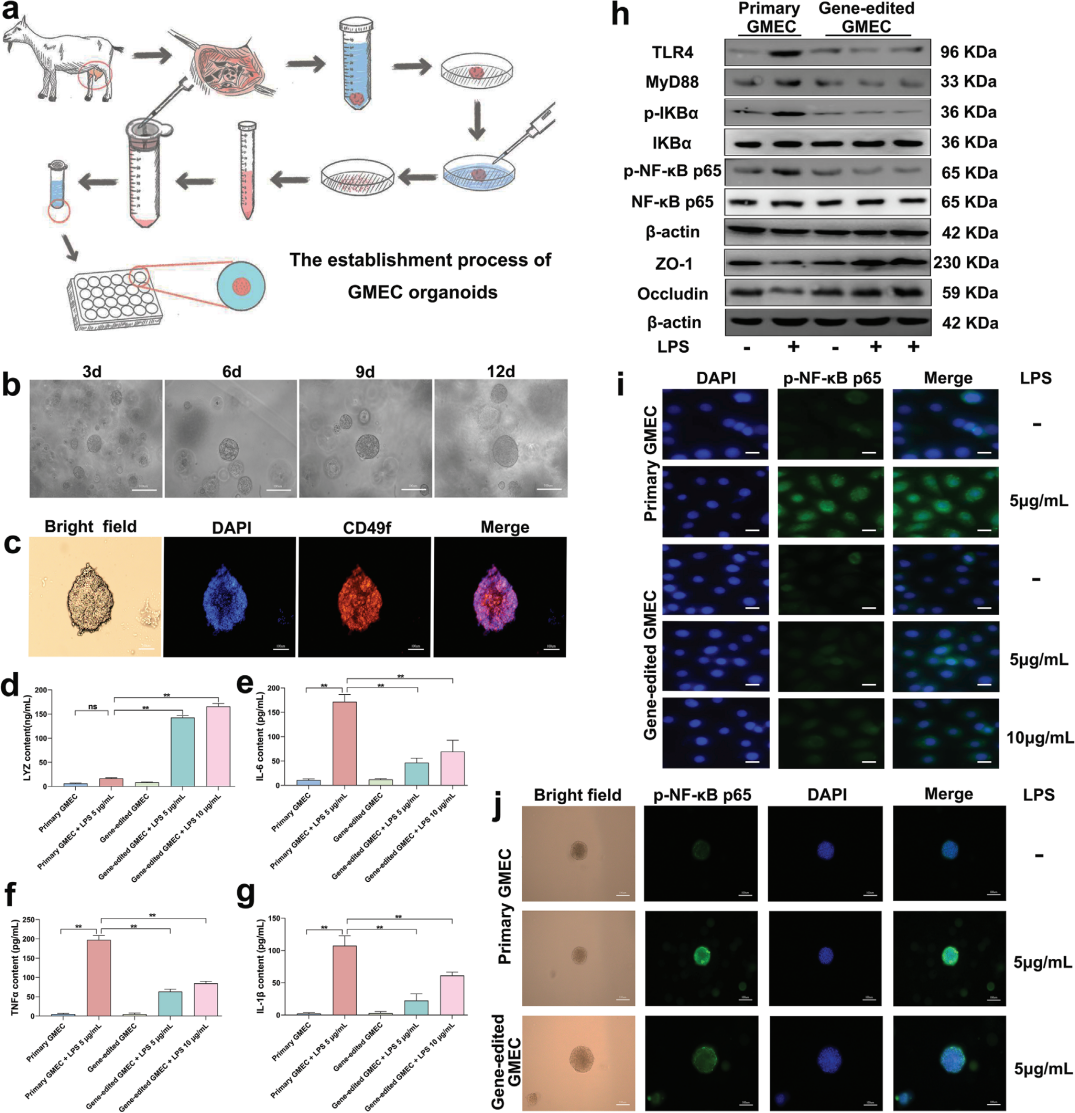
Gene‐edited GMEC alleviates inflammation and TJ damage by high expression of LYZ. a) Flowchart of GMEC organoid establishment. b) GMEC organoid culture 3, 6, 9 and 12 d status. Scale bar: 100 µm. c) CD49f staining of GMEC organoids was performed using the IF method. Scale bar: 100 µm. d–g) Analysis of LYZ, IL‐6, TNFα, IL‐1β protein expression. h) Primary GMEC and gene‐edited GMEC were treated with 5 and 10 µg mL^−1^ LPS for 12 h, and then the culture medium was replaced for 12 h to detect the protein expression level of TLR4, MyD88, p‐IKBα, IKBα, p‐NF‐κB‐p65, NF‐κB‐p65, ZO‐1 and Occludin. i) The protein expression of p‐NF‐κB‐p65 in the nucleus was detected by IF. Scale bar: 20 µm. j) The protein expression of p‐NF‐κB p65 in GMEC organoids was detected by IF. Scale bar: 100 µm. Values are expressed as mean ± SEM (n = 3 per group) by one‐way ANOVA. *: indicates significant difference (*P* < 0.05), **: indicates that the difference is highly significant (*P* < 0.01) and ns: indicates no significant difference (*P* > 0.05).

In vivo experiments, we found that the high expression of LYZ in GED goats can alleviate mastitis and BMB damage very well. However, LYZ exerts the anti‐inflammatory effect and alleviates BMB damage needs to be further analyzed. We isolated gene‐edited GMEC from the mammary gland of GED goats for experimental validation and established a gene‐edited GMEC organoid model to accurately assess the anti‐inflammatory effects and the mechanism of mitigate BMB damage within the mammary gland of GED goats. The establishment process of GMEC organoids is shown in the figure (**Figure** **6**a,b). The results showed that integrin subunit alpha 6 (CD49f) protein was expressed in GMEC organoids (Figure 6c), and l*eucine rich repeat containing g protein‐coupled receptor 5* (*LGR5*), *sry‐box transcription factor 2* (*SOX2*), and *sry‐box transcription factor 9* (*SOX9*) mRNA were significantly expressed in GMEC organoids (Figure S13, Supporting Information), indicating that GMEC organoids presence of stem cell characteristics with cellular clustering and self‐renewal potential.^[^ ^14^ ^]^ Therefore, this model was used for subsequent experimental validation.

Subsequently, an in vitro model of mastitis GMEC was established using LPS, and primary GMEC and gene‐edited GMEC were treated with different concentrations of LPS for 12 h, and subsequently cultured with fresh medium for 12 h. Results indicated that compared to primary GMEC treated with LPS, the gene‐edited GMEC treated with LPS showed significantly increased levels of LYZ mRNA and protein expression (Figure 6d; Figure S14a, Supporting Information). Additionally, mRNA and protein expression levels of TLR4, MyD88, p‐IKBα, p‐NF‐κB p65, IL‐6, TNFα and IL‐1β were significantly decreased (Figure 6e–h; Figure S14b–h and Figure S15, Supporting Information). The expressions of ZO‐1 and Occludin protein were significantly increased (Figure 6h; Figure S16a,b, Supporting Information), and the mRNA expression level was consistent with that of protein (Figure S16c,d, Supporting Information). In addition, the transfer of p‐NF‐κB p65 protein to the nucleus was inhibited, and consistent with the GMEC organoid results (Figure 6i,j) and the stable distribution of ZO‐1 and Occludin on the cell membrane was protected, the integrity of TJ was maintained, consistent with the GMEC organoid results (Figure S16e–h, Supporting Information). These results indicate that gene‐edited GMEC with high expression of LYZ could play an anti‐inflammatory role, inhibit the activation of TLR4/MyD88/NF‐κB, prevent p‐NF‐κB p65 transfer to the nucleus, reduce the expression of inflammatory factors, alleviate the inflammatory response of GMEC, and protect the integrity of TJ. This also corroborated the in vivo experimental results showing that dairy goat LYZ can exerts a favorable anti‐inflammatory effect and alleviates BMB damage. However, how LYZ alleviates BMB damage caused by mastitis must be further demonstrated.

### 2.6 Dairy Goat LYZ Exerts Anti‐inflammatory Effects by Downregulation of HMGB1 Expression in Gene‐edited GMEC

**Figure 7:**
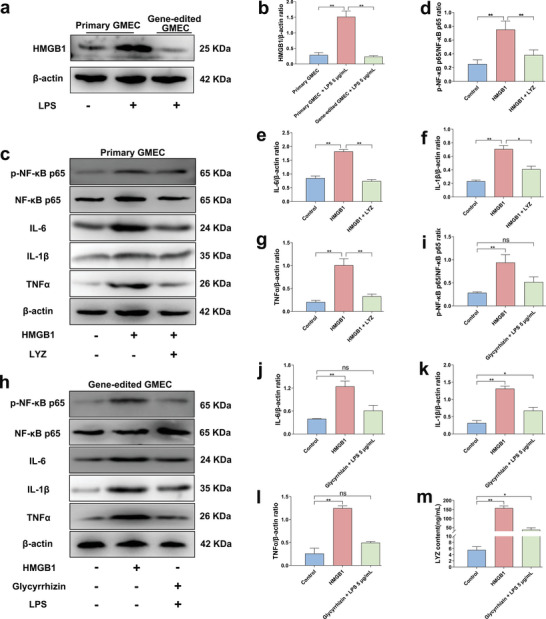
Dairy goat LYZ exerts anti‐inflammatory effects by down‐regulation of HMGB1 expression in gene‐edited GMEC. a) Primary GMEC and gene‐edited GMEC were treated with 5 µg mL^−1^ LPS for 12 h, and then the culture medium was replaced for 12 h to detect the protein expression level of HMGB1. b) The protein ratio of HMGB1 and β‐actin. c) Primary GMEC was treated with 1 µg mL^−1^ HMGB1 recombinant protein followed by treatment with 10 nM dairy goat LYZ recombinant protein for 12 h to detect the protein expression levels of p‐NF‐κB‐p65, NF‐κB‐p65, IL‐6, TNFα, and IL‐1β. d) The protein ratio of p‐NF‐κB‐p65 and NF‐κB‐p65. e–g) The protein ratio of IL‐6, TNFα, IL‐1β and β‐actin. h) Gene‐edited GMEC was treated with 1 µg mL^−1^ HMGB1 recombinant protein, 40 µM Glyeyrrhizin and 5 µg mL^−1^ LPS to detect the protein expression levels of p‐NF‐κB‐p65, NF‐κB‐p65, IL‐6, TNFα, and IL‐1β. i) The protein ratio of p‐NF‐κB‐p65 and NF‐κB‐p65. j–l) The protein ratio of IL‐6, TNFα, IL‐1β and β‐actin. m) Analysis of LYZ protein expression. Values are expressed as mean ± SEM (n = 3 per group) by one‐way ANOVA. *: indicates significant difference (*P* < 0.05), **: indicates that the difference is highly significant (*P* < 0.01) and ns: indicates no significant difference (*P* > 0.05).

HMGB1 is an important factor in the inflammatory process and a therapeutic target for mitigating inflammatory responses.^[^ ^15^ ^]^ In the heatmap results, *HMGB1* expression was significantly altered during LYZ alleviation of the inflammatory response in GMEC (Figure 1e). Therefore, we isolated gene‐edited GMEC from the mammary glands of GED goats for experimental validation to determine the mechanism of the resistance in GED goats to mastitis with high LYZ expression and exploring whether gene‐edited GMEC exerts a potential anti‐inflammatory effect by modulating HMGB1 expression following elevated levels of LYZ. Both primary GMEC and gene‐edited GMEC were treated with 5 µg mL^−1^ LPS for 12 h, followed by culturing with fresh medium for 12 h. These findings revealed a notable increase in HMGB1 expression in primary GMEC following LPS treatment. In contrast, gene‐edited GMEC exhibited significant inhibition of HMGB1 expression in response to LPS, indicating a regulatory effect of dairy goat LYZ on HMGB1 expression (**Figure** **7**a,b; Figure S17a, Supporting Information).

Subsequently, after a 12 h treatment of primary GMEC with 1 µg mL^−1^ of HMGB1 recombinant protein, dairy goat LYZ recombinant protein was administered at a concentration of 10 nM for 12 h to investigate its potential in alleviating the inflammatory response induced by HMGB1. The findings revealed that HMGB1 markedly increased the expression levels of p‐NF‐κB p65, IL‐6, TNFα and IL‐1β mRNA and protein, inducing inflammation in primary GMEC. However, upon the addition of dairy goat LYZ recombinant protein, both mRNA and protein expression levels of p‐NF‐κB p65, IL‐6, TNFα, and IL‐1β were significantly decreased, suggesting that dairy goat LYZ could mitigate the inflammatory response in GMEC induced by HMGB1 (Figure 7c–g; Figure S17b–e, Supporting Information).

Finally, gene‐edited GMEC were isolated from the mammary glands of GED goats for experimental validation. The gene‐edited GMEC were pretreated with 40 µM Glycyrrhizin (HMGB1 inhibitor) for 2 h and then treated with 5 µg mL^−1^ LPS for 12 h; the treatment duration with 1 µg mL^−1^ recombinant HMGB1 protein was continued until the end of the experiment. The aim was to investigate the effect of changes in HMGB1 expression on the inflammatory responses in gene‐edited GMEC. The results showed that continuous HMGB1 treatment could significantly increase the expression levels of p‐NF‐κB p65, IL‐6, TNFα, and IL‐1β mRNA and protein, thereby eliciting an inflammatory response in gene‐edited GMEC (Figure 7h,i; Figure S17f–i, Supporting Information). HMGB1 treatment also notably increased LYZ mRNA and protein expression levels (Figure 7m; Figure S17j, Supporting Information); however, it did not alleviate the inflammatory response in gene‐edited GMEC. These findings suggest that the prolonged presence of HMGB1 exacerbates inflammation in gene‐edited GMEC, and despite the elevated expression of LYZ, it was unable to reduce HMGB1 expression owing to the persistent presence of HMGB1. However, compared to the control group, the gene‐edited GMEC were pretreated with Glycyrrhizin for 2 h and then treated with LPS for 12 h, the expression of p‐NF‐κB p65, IL‐6, TNFα, and IL‐1β mRNA and protein was significantly inhibited, while the expression of LYZ was significantly elevated but not as much as after the addition of HMGB1 alone (Figure 7h–m; Figure S17f–j, Supporting Information). These results indicated that inhibiting the expression of HMGB1 could reduce the inflammatory response in gene‐edited GMEC.

In summary, HMGB1 is a key factor in the inflammatory response, and LYZ regulates the expression of HMGB1, thereby alleviating the inflammatory response. Therefore, LYZ can regulate the inflammatory response by down‐regulating the expression of HMGB1 in gene‐edited GMEC, which is one of the regulatory mechanisms of the anti‐mastitis in vivo following high LYZ expression in GED goats.

### 2.7 Gene‐Edited GMEC Inhibits Activation of PANoptosis and Antagonize TJ Damage by High Expression of LYZ

In this study, heatmap analysis results showed that LYZ can downregulate the expression of major genes involved in PANoptosis in an in vitro GMEC mastitis model (Figure 1e), and the in vivo results demonstrated that high expression of LYZ can alleviate BMB damage in GED goats; however, the detailed mechanism by which mastitis leads to BMB damage is not clear. In mouse models of pneumonia, there is a direct relationship between PANoptosis activation and lung barrier damage.^[^ ^16^ ^]^ Therefore, it is necessary to explore whether the activation of PANoptosis can be inhibited after high expression of LYZ in GED goats when mastitis occurs. We hypothesized whether combined inhibition of PANoptosis components could induce TJ damage in the GMEC mastitis model to determine the mechanism of BMB damage in vivo.

**Figure 8:**
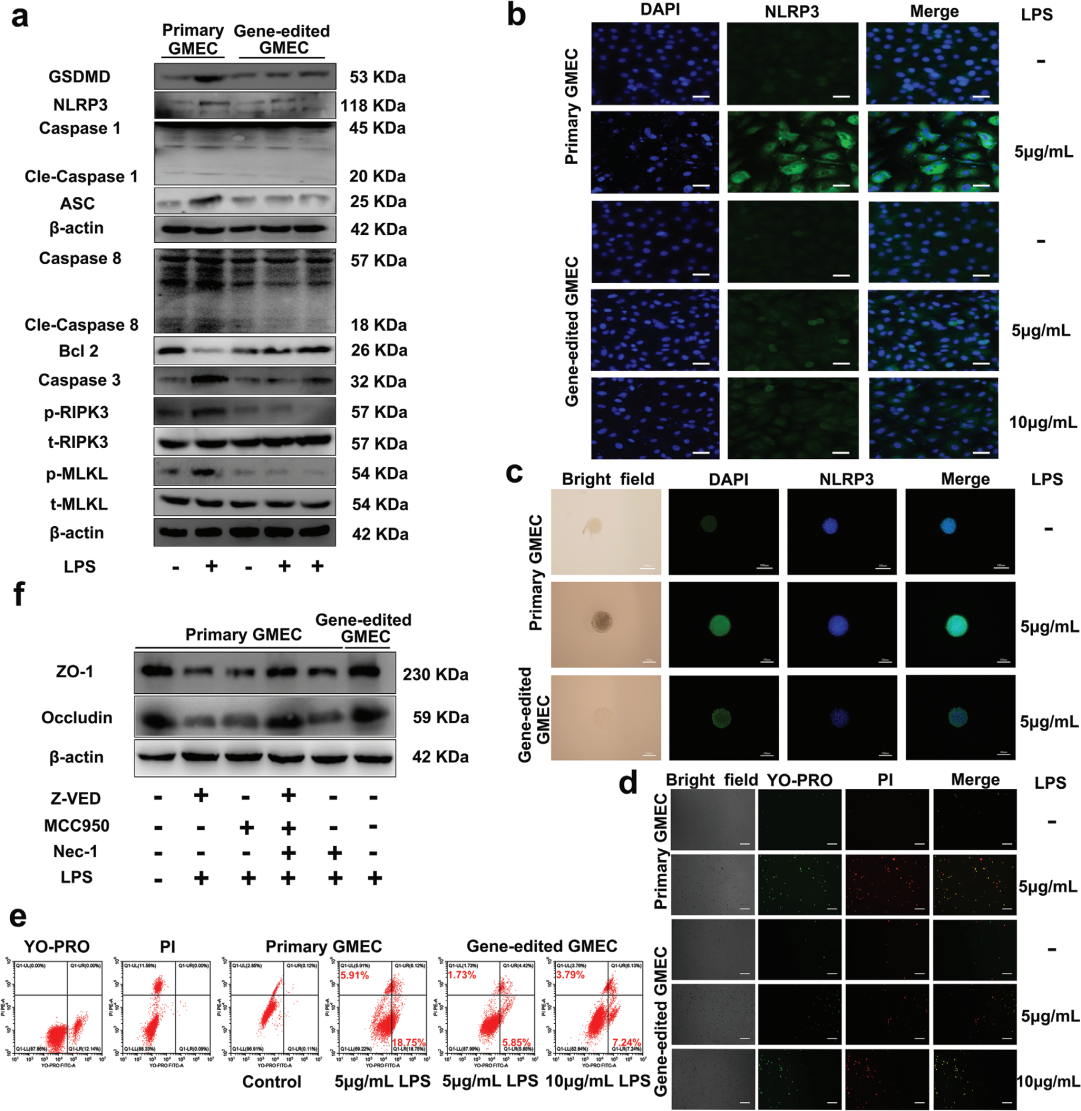
Gene‐edited GMEC inhibits PANoptosis activation by high expression of LYZ. a) Primary GMEC and gene‐edited GMEC were treated with 5 µg mL^−1^ LPS and 10 µg mL^−1^ LPS for 12 h, and then the culture medium was replaced for 12 h to detect the protein expression levels of GSDMD, NLRP3, Caspase 1, Cle‐Caspase 1, ASC, Caspase 8, Cle‐Caspase 8, Bcl2, Caspase 3, p‐RIPK3, t‐RIPK3, p‐MLKL and t‐MLKL. b) The protein expression of NLRP3 in GMEC was detected by IF. Scale bar: 50 µm. c) The protein expression of NLRP3 in GMEC organoids was detected by IF. Scale bar: 100 µm. d) Primary GMEC and gene‐edited GMEC were treated with 5 µg mL^−1^ LPS and 10 µg mL^−1^ LPS for 12 h, and then the culture medium was replaced for 12 h to detect the proportion of apoptosis and necrosis (n = 3 per group). Scale bar: 100 µm. e) Primary GMEC and gene‐edited GMEC were treated with 5 µg mL^−1^ LPS and 10 µg mL^−1^ LPS for 12 h, and then the culture medium was replaced for 12 h to detect apoptosis ratio (n = 3 per group). f) Under inflammatory conditions, primary GMEC were treated with Z‐VED, MCC950 and Nec‐1 inhibitors to inhibit the activation of PANoptosis and to detect the protein expression of ZO‐1 and Occludin.

For experimental validation, we isolated gene‐edited GMEC from the mammary glands of GED goats. Primary GMEC and gene‐edited GMEC were treated with different concentrations of LPS for 12 h, and subsequently cultured with fresh medium for 12 h. Results indicated that compared to primary GMEC treated with LPS, the gene‐edited GMEC treated with LPS showed significantly decreases in the expression levels of GSDMD, NLRP3, Cle‐Caspase 1, ASC, Cle‐Caspase 8, Caspase 3, p‐RIPK3 and p‐MLKL proteins, while the expression levels of Bcl2 protein were significantly increased (**Figure** **8**a; Figure S18, Supporting Information). The mRNA expression levels were consistent with that of those protein (Figure S19, Supporting Information). Moreover, the expression of NLRP3 protein in cells was markedly reduced (Figure 8b), consistent with the GMEC organoid results (Figure 8c). This finding is also consistent with the results of the heatmap analysis, indicating that the high LYZ expression has a regulatory effect on the expression of PANoptosis‐related genes (Figure 1e). In the YO‐PRO‐1/PI staining experiment, the proportion of green‐positive cells (apoptosis) and red‐positive cells (necrosis) was significantly decreased in gene‐edited GMEC treated with LPS compared with primary GMEC treated with LPS (Figure 8d). Furthermore, flow cytometry analysis revealed that the apoptosis ratio of gene‐edited GMEC decrease from 18.75% to 5.85% and 7.24%, respectively (Figure 8e). In summary, PANoptosis was activated in the in vitro LPS‐induced GMEC mastitis model. However, high LYZ expression in gene‐edited GMEC can inhibit the activation of PANoptosis. This suggested that high expression of LYZ in GED goats can inhibit PANoptosis activation when mastitis occurs.

Finally, primary GMEC were pre‐treated with Z‐VAD‐FMK (Z‐VAD, Caspase inhibitor, 50 µM for 2 h), MCC950 (NLRP3 inhibitor, 50 µM for 2 h) and Necrostatin‐1 (Nec‐1, RIPK inhibitor, 50 µM for 2 h), followed by treatment with 5 µg mL^−1^ LPS for 12 h and culturing with fresh medium for 12 h. The results revealed that primary GMEC significantly reduced the mRNA and protein expression of ZO‐1 and Occludin compared to the control group after treatment with Z‐VAD, MCC950 and Nec‐1, respectively (Figure 8f; Figure S20, Supporting Information). Notably, there were no significant differences in ZO‐1 and Occludin mRNA and protein expression in primary GMEC co‐treated with Z‐VAD, MCC950, Nec‐1, and LPS compared to those in the control group, which was attributed to the inhibition of PANoptosis activation (Figure 8f; Figure S20, Supporting Information). Furthermore, we isolated gene‐edited GMEC from the mammary gland of GED goats for experimental validation. There were no significant differences in ZO‐1 and Occludin mRNA and protein expression after treatment of gene‐edited GEMC followed by treatment with 5 µg mL^−1^ LPS for 12 h and culturing with fresh medium for 12 h, compared to the control group (Figure 8f; Figure S20, Supporting Information). In summary, these results showed that in the case of inflammation, inhibition of PANoptosis activation using an inhibitor is effective in preventing the reduction of TJ protein expression in GMEC; the high expression of LYZ in gene‐edited GMEC prevents this phenomenon, indicating that LYZ can inhibit PANoptosis activation in the presence of inflammation, thereby mitigating TJ damage in GMEC. This is one of the main mechanism to alleviate BMB damage in vivo after high expression of LYZ in GED goats.

## 3 Discussion

The clinical use of antibiotics to treat mastitis often leaves behind drug residues and contributes to the development of bacterial resistance, which affects both human and animal health.^[^ ^17^ ^]^ At present, a variety of anti‐mastitis transgenic cattle and goats have been created through gene‐editing technology by enhancing the expression of foreign genes such as defensins and lysostaphin in milk, the anti‐mastitis capabilities of these animals have been substantially improved.^[^ ^2^, ^10^, ^18^ ^]^ However, because of the complex safety evaluation system of transgenic technology, it is difficult to apply transgenic animals in the livestock industry. Therefore, it is particularly important to create safer and more effective non‐transgenic mastitis‐resistant animals.^[^ ^19^ ^]^ In previous studies, CRISPR‐associated enzymes have been used extensively to produce gene‐edited animals, but their large size limits their applicability, especially in eukaryotic gene‐editing, where larger Cas proteins are difficult to deliver into target cells; however, the emergence of the ISDra2‐TnpB system fills this gap.^[^ ^3^, ^4^, ^20^ ^]^ Therefore, in this study, IRS was integrated into the *LYZ* promoter region using the ISDra2‐TnpB system, and GED goats were obtained by utilizing SCNT, which verified the feasibility of a regulatory sequence gene editing breeding strategy for large animals, providing a new editing strategy for future breeding of animal diseases. Importantly, we believe that this strategy is applicable to livestock, and can be broadly applied.

In the absence of mastitis, LYZ expression was no significant changed in the mammary glands of GED goats. However, under mastitis conditions, LYZ expression was increased by IRS, thereby reducing the severity of mastitis and showing robust anti‐mastitis capabilities. Previous studies have shown that increasing LYZ expression in the mammary gland effectively mitigates mastitis disease and maintains homeostasis of the immune environment.^[^ ^10^, ^21^ ^]^ Furthermore, LYZ attenuated the intestinal inflammatory response and improved the intestinal immune environment in piglets and mice.^[^ ^11^ ^]^ Therefore, increasing LYZ expression in the mammary glands is an effective strategy to treat mastitis. It has been reported that the mRNA expression level of *TLR4* in cows with subclinical mastitis is 7.49 times higher than that in cows without the disease.^[^ ^22^ ^]^ We speculated that the presence of an IRS within the *TLR4* promoter region, leads to elevated TLR4 expression levels under inflammatory conditions. Therefore, we performed functional verification by integrating the screened IRS into the promoter region of *LYZ* using the ISDra2‐TnpB system and found that IRS could enhance the expression of LYZ in vivo and in vitro under inflammatory conditions. In addition, eradicating mastitis not only involves the removal of the pathogenic bacteria, but also the elimination of inflammation within the mammary gland in order to maintain the homeostasis of the immune environment. Although goat LYZ shares high homology with human LYZ, its functional similarity remains unknown. Therefore, a GMEC mastitis model was established in vitro, and RNA‐seq analysis was performed after dairy goat LYZ treatment. These findings revealed that dairy goat LYZ treatment led reduced pro‐inflammatory factors and immune pathway activation, suggesting its potential to alleviate GMEC inflammatory responses. Further validation using in vivo and in vitro experiments showed that IRS enhanced LYZ expression and mitigated the inflammatory response in gene‐edited GMEC and GED goats in the mammary gland, highlighting the anti‐inflammatory effects of dairy goat LYZ. Previous studies have shown that human LYZ inhibits LPS‐induced inflammation in human endothelial cell and mouse sepsis by regulating the expression of HMGB1.^[^ ^15^ ^]^ More importantly, HMGB1 is associated with mastitis disease, and binds to receptors such as TLR2 and TLR4 to enhance the inflammatory response.^[^ ^23^ ^]^ However, whether dairy goat LYZ exerts its anti‐inflammatory effects by regulating the expression level of HMGB1 is unknown, which we verified. The results showed that the presence of HMGB1 could indeed aggravated the inflammatory response in GMEC, and this phenomenon could be improved by adding recombinant dairy goat LYZ protein. Moreover, HMGB1 expression was inhibited under the inflammatory conditions in gene‐edited GMEC, indicating that the high expression of LYZ reduced the expression of HMGB1 and alleviated the inflammatory response. This is one of the regulatory mechanisms of the anti‐mastitis in GED goats with high LYZ expression.

High LYZ expression in GED goats reduced the severity of mastitis and alleviated BMB damage. Recently, the integrity and permeability of the BMB have emerged as crucial considerations in mastitis research because alleviating BMB damage can impede the spread of mastitis.^[^ ^7^, ^24^ ^]^ This study proved that GED goats with high LYZ expression could alleviate BMB damage, effectively protect the integrity of the structure within the mammary gland, and maintain the dynamic balance of inflammatory factors, preventing the further mastitis expansion. In addition, TJ play a critical role in the formation of the main structure of the BMB.^[^ ^25^ ^]^ Previous studies have shown that LPS treatment induces inflammation in Sertoli cells, leading to TJ damage, which be ameliorated by inhibiting inflammation.^[^ ^25b^ ^]^ In this study, the elevated LYZ expression effectively alleviated the onset of inflammatory responses in GMEC, thereby safeguarding the integrity of TJ. However, the mechanism by which inflammation leads to TJ damage is not well understood. TJ proteins undergo proteolytic cleavage after apoptosis, thereby disrupting cell integrity and permeability.^[^ ^26^ ^]^ Recent research indicates that bacteria via Toll‐Like Receptors, induce the production of pro‐inflammatory cytokines that promote inflammatory signaling, PANoptosis activation, the subsequent release of numerous inflammatory mediators, and cell death, thus amplifying the inflammatory response.^[^ ^27^ ^]^ In a mouse model of lung damage, the activation of PANoptosis exacerbated lung inflammation and disrupted of the respiratory barrier.^[^ ^28^ ^]^ Therefore, we hypothesized and validated that the main cause of BMB damage at the onset of mastitis was the activation of PANoptosis. Notably, in an in vitro GMEC mastitis model, gene‐edited GEMC with high LYZ expression decreased the expression of key PANoptosis proteins and significantly reduced the rates of apoptosis and cell necrosis, suggesting that LYZ effectively inhibited the activation of PANoptosis. In addition, the activation of PANoptosis is related to the release of HMGB1.^[^ ^29^ ^]^ This, combined with the experimental results of this study, suggested that dairy goat LYZ is closely associated with the inhibition of PANoptosis activation by down‐regulating the expression of HMGB1. Recent studies have found that GSDMD activation is a major protein, not an inflammatory factor, in inflammatory Blood‐brain barrier breakdown.^[^ ^30^ ^]^ More importantly, GSDMD is a key protein involved in the PANoptosis. This study verified that inhibition of the PANoptosis pathway could effectively prevent the decline in TJ protein expression, suggesting that dairy goat LYZ mitigates TJ damage by inhibiting the PANoptosis pathway, which is the pathways in the GED goats with high LYZ expression can effectively mitigate BMB damage. Therefore, high LYZ expression in GED goats can improve the permeability and integrity of the BMB by inhibiting the activation of PANoptosis and preventing the release of pro‐inflammatory factors, thereby preventing the progression of mastitis.

In summary, this study successfully generated GED goats using the ISDra2‐TnpB system and demonstrated its efficacy in resisting *E. coli*‐induced mastitis. GED goats exhibit an innate capacity for high LYZ expression, thus conferring safe, efficient, and specific anti‐mastitis properties. Crucially, the designed gene‐editing strategy avoids the introduction of foreign genes and significantly improves its biosafety, making it safer and more effective than using transgenic animals. This study provided the initial evidence implicating PANoptosis in mastitis progression and BMB damage and suggested that it is a potential therapeutic target. Additionally, our findings suggested that the anti‐inflammatory action of LYZ can be achieved by regulating HMGB1 expression, thereby mitigating the progression of mastitis. This study is important because it enhanced biosafety, introduced a novel and efficient editing approach for developing disease‐resistant animals through gene‐editing technology, while providing a research basis for the widespread application of the ISDra2‐TnpB system.

## 4 Conclusion

**Figure 9:**
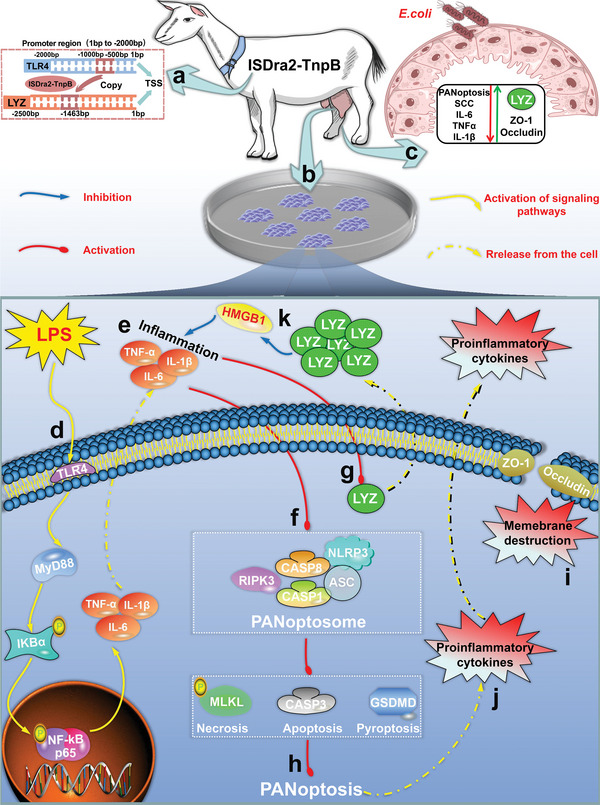
A overview chart for mechanism of alleviating mastitis and BMB damage by GED goats with high expression of LYZ. a) According to the regulatory sequence gene editing breeding strategy proposed in this study, GED goats were created by targeted integration of IRS into the *LYZ* promoter region using the ISDar2‐TnpB system. b) Isolation of GMEC from GED goats for culture and establishment of GMEC organoids. c) *E.coli* infection of the mammary gland of GED goats increases LYZ expression, which inhibits the activation of PANoptosis, decreases SCC and proinflammatory factor expression in milk, and increases ZO‐1 and occludin expression, thereby alleviating mastitis severity and BMB damage. d) In vitro, a mastitis model was established by treating GMEC with LPS, and the TLR4/MyD88/NF‐κB signaling pathway was activated. e) When the TLR4/MyD88/NF‐κB signaling pathway is activated, a large amount of pro‐inflammatory factors are produced and secreted extracellularly. f) In an in vitro GMEC mastitis model, the PANoptosome can be activated. g) Under inflammatory conditions, IRS upregulates LYZ expression in gene‐edited GMEC. h) In an in vitro GMEC mastitis model, the PANoptosis can be activated. i) Activation of PANoptosis disrupts membrane integrity and permeability. j) Activation of PANoptosis results in the release of a large number of pro‐inflammatory factors. k) In an in vitro GMEC mastitis model, LYZ can exert an anti‐inflammatory role by regulating the expression of HMGB1.

In this study, we proposed a regulatory sequence gene‐editing breeding strategy and verified its feasibility by creating mastitis‐resistant GED goats using the ISDra2‐TnpB system. Upon *E. coli* mammary gland infection, GED goats exhibited significantly increased LYZ expression, thereby inhibiting the activation of PANoptosis, alleviating BMB damage and reducing the severity of mastitis (**Figure** **9**).

## 5 Experimental Section

### Ethics Statement

All animal procedures conducted at Northwest Agriculture & Forestry University were approved by the Animal Ethics Committee and adhered strictly to the “Guidelines for Animal Care and Use for Research Purposes.” Efforts were made to minimize animal pain, suffering, and distress, as well as to reduce the number of animals used. Additionally, all surgeries were performed under anesthesia induced by intravenous injection of Sumianxing (Veterinary Research Institute, Jilin, China).

### Cell Culture and Treatment

The experimental cells included GFFs and GMEC, both of which were derived from lactating Saanen dairy goats, and  GMEC from GED goats, which were isolated and cultured according to previous methods.^[^ ^31^ ^]^ The HEK293T cells were cultured in Gibco fetal bovine serum (9%), penicillin‐streptomycin (1%), and Gibco DMEM medium (90%). During the experiments, all primary GMEC and gene‐edited GMEC were pretreated with LPS, followed by three washes with PBS and subsequent culture with fresh medium. This was used to exclude that the combination of LYZ and LPS affected the experimental results. HMGB1, Z‐VED, MCC950, Ner‐1, and Glyeyrrhizin for cell treatment were procured from MCE (MedChemExpress, USA). Unless otherwise indicated, LPS (L2880, Sigma, USA) concentrations of 0.25, 0.5, 1, 2.5, 5, and 10 µg mL^−1^ were used in the experiment, while LYZ recombinant protein concentrations for dairy goats (NP_0 012 72640.1) (Sino Biological inc, Beijing, China) were 1, 2.5, 5, 10, and 20 nM.

### CCK8 Assay

A 96‐well plate was used, with each well receiving 200 µL of a cell suspension containing 2 × 10^4^ cells mL^−1^ in serum‐containing medium. Following incubation at 37 °C in a 5% CO_2_ humidified environment for 6, 12, 24 and 48 h, 10 µL of CCK8 (96992, Sigma, USA) solution was added to each well, and OD was measured at 450 nm using a microplate reader to assess cell viability.

### Dual‐Luciferase Reporter System

TransDetect Double‐Luciferase Reporter Assay Kit purchased from TransGen Biotech (Beijing, China) was used in strict adherence to the manufacturer's instructions. In this study, a dual‐luciferase reporter system was utilized to screen for IRS. The promoter region (1 bp to −2000 bp) of *TLR4* (NCBI ID: 100860955) was truncated and cloned into the pGL4.10 vector, and generate constructs designated as pGL4.10‐1/−500 (TSS 1 bp to ‐500 bp), pGL4.10‐1/−1000 (TSS 1 bp to −1000 bp), pGL4.10‐1/−1500 (TSS 1 bp to −1500 bp), and pGL4.10‐1/−2000 (TSS 1 bp to −2000 bp). The *LYZ* (NCBI ID: 100 860 864) promoter region sequence was amplified by PCR and subsequently cloned into the pGL4.10 vector (pGL4.10‐LYZ). Subsequently, sequences containing a combination of the TSS −500 to −1000 bp region and the *LYZ* promoter region were synthesized by Sangon Biotech (Shanghai, China), and subsequently cloned into the pGL4.10 vector (pGL4.10‐IRS‐LYZ). The vectors were transfected into HEK293T cells and GMEC using Lipofectamine 3000 (L3000015, Thermo Fisher Scientific inc, USA), and treated with LPS. Finally, the firefly luciferase reporter gene and sea kidney luciferase reporter gene were detected by the dual‐luciferase reporter system, and calculate promoter activity. Table S1 (Supporting Information) below lists the detailed information of the primers.

### Vector Construction

The targeting vector (pRZ122) utilized in this study was derived from previously reported plasmids.^[^ ^4b^ ^]^ The TnpB protein and reRNA (Contains 20 bp target sequence next to the TAM) were synthesized by Sangon Biotech (Shanghai, China) and cloned into the PX458 vector (Addgene #48 138) under the CAG and U6 promoters, respectively. Detailed sequence information in supplementary Table S2. The donor vector comprised a pMD19‐T vector (6013, Takara Bio Inc., China) skeleton, with left and right homologous arms of 800 bp upstream and downstream of the sgRNA, respectively. The LOXP‐EF‐1α‐EGFP‐P2A‐PuroR‐SV40 poly(A)‐LOXP sequence was synthesized by Sangon (Shanghai, China), and the IRS was obtained through PCR amplification of the genome. The above fragments were cloned into the vector skeleton using homologous recombination. Detailed information regarding the primers used was provided in Table S1 (Supporting Information).

### Screening for SgRNA

The sgRNAs were designed to target the region spanning from the *LYZ* promoter TSS ‐500 to ‐2000 bp, based on TAM sequences (TTGAT and ATCAA). Six sgRNAs were selected for target efficiency assessment. Constructs pRZ122/sgRNA1, pRZ122/sgRNA2, pRZ122/sgRNA3, pRZ122/sgRNA4, pRZ122/sgRNA5 and pRZ122/sgRNA6 were generated based on the pRZ122 plasmid. The cleavage activity of sgRNAs were evaluated using the SSA method.^[^ ^32^ ^]^ The SSA reporter plasmids (pSSA‐sgRNA1, pSSA‐sgRNA2, pSSA‐sgRNA3, pSSA‐sgRNA4, pSSA‐sgRNA5 and pSSA‐sgRNA6) were constructed by inserting the six different target sites into the pSSA‐1‐3 reporter plasmid. Briefly, the pSSA‐1‐3 vector universal primer RvP3 was used as the upstream primer, and the synthetic primers containing sgRNAs were used as the downstream primers (SSA‐SgRNA1‐R, SSA‐SgRNA2‐R, SSA‐SgRNA3‐R, and SSA‐SgRNA4‐R), and PCR was performed using the pGL3‐Control vector as the template for amplification, and cloned into the pMD19‐T vector (6013, Takara Bio Inc., China). The above obtained vector and the pSSA‐1‐3 vector were subjected to BglII (1021B, Takara Bio Inc., China) and EcoRI (1040B, Takara Bio Inc., China) double‐enzymatic cleavage, respectively, followed by ligation of the double‐enzymatically cleaved target fragment into the double‐enzymatically cleaved pSSA‐1‐3 backbone vector to construct the pSSA‐sgRNA vector, which was finally identified using PCR amplification using LucRep‐F and LucRep‐R. Finally, HEK293T cells were cultured and seeded into 24‐well plates until reaching 90% confluence for transfection. The transfection system involved combining 0.6 µg of pRZ122/sgRNA plasmid, 0.18 µg of pSSA‐sgRNA plasmid, 0.02 µg of pRL‐SV40, and 1 µL of P3000 reagent (L3000015, Thermo Fisher Scientific inc, USA) in a 50 µL Opti‐MEM tube per well, followed by mixing with an additional 50 µL of Opti‐MEM containing 0.75 µL of Lipofectamine 3000 (L3000015, Thermo Fisher Scientific inc, USA). After incubating at room temperature for 10 min, the transfection solution was gently added to the labeled 24‐well plate, and cells were cultured in a 5% CO_2_ incubator at 37 °C for 8–10 h before changing the medium. Samples were collected after 48 h and analyzed using the dual‐luciferase reporter system, and data were analyzed to select the most effective sgRNA. SgRNAs for CRISPR/Cas9 were designed according to the CRISPOR website (http://crispor.tefor.net/), using the same approach for cleavage activity assessment. Detailed information regarding the primers used was provided in Table S1 (Supporting Information).

### Screening and Identification of Gene‐edited Cells

Cells were seeded into 60 mm dishes and passaged when reaching 90% confluence. Upon reaching optimal growth, cells from all dishes were harvested and combined with the electroporation buffer. The electroporation buffer consisted of 600 µL Opti‐MEM (31 985 070, Thermo Fisher Scientific inc, USA) 200 µL, the pRZ122/sgRNA4 vector (4 µg), and the donor vector (6 µg), mixed in a 1.5 mL centrifuge tube. The mixture was gently mixed and transferred into the BTX electric cup (ECM2001, BTX Technologies, USA), allowing it to stand for 10 min. Electroporation was conducted at 510 V voltage, 2 ms pulse, and 1–2 shocks. Following electroporation, the cell suspension was evenly distributed into two 100 mm petri dishes, supplemented with culture medium, and returned to the incubator for further culture. After 48 h of culture, the medium was replaced with fresh medium containing 2 µg mL^−1^ Puromycin (ST551, Beyotime, China) for drug screening, and the cell growth status was observed every 2–3 d. After 7 d of purinomycin screening, positive monoclonal cells with green fluorescence were observed using fluorescence microscope. Subsequently, positive monoclonal cells were selected under an inverted stereo microscope, numbered, and seeded into 48‐well plates for further analysis. Approximately 15% of the selected cells were subjected to junction PCR identification. Upstream primers matching the genome sequence 500 bp upstream of the 5ʹ end homologous arm and downstream primers targeting the EGFP sequence were used for 5ʹ‐junction PCR, yielding a product size of 3000 bp. For 3ʹ‐junction PCR, upstream primers matched the puromycin sequence, and downstream primers targeted the genome sequence 500 bp downstream of the 3ʹ end homologous arm, resulting in a product size of 2200 bp. Table S1 (Supporting Information) below lists the detailed information of the primers.

Unlabeled positive monoclonal cell clusters were obtained by electrotransfecting green fluorescent positive monoclonal cell clusters with the pcDNA3‐Cre vector (V012087#, NovoPro Bio Inc., China). The electrotransfection conditions were consistent with the previously described ones. After the electrotransfection, cells were supplemented with culture medium and returned to the incubator for further growth. Once cells reached ≈90% confluence, they were sorted using flow cytometry. Sorted cells were then seeded into 48‐well plates and cultured until reaching ≈90% confluence for PCR identification. The upstream primers were designed to match the upstream genomic sequence of the sgRNA4 at 500 bp position, and the downstream primers were designed to match the downstream genomic sequence at 500 bp position of the sgRNA4. The expected PCR product size was 1534 bp. Table S1 (Supporting Information) below lists the detailed information of the primers.

### Production of GED Goats

Gene‐edited GFFs were seeded onto a 48‐well plate, and allowed to reach confluence over a period of 1–2 d prior to SCNT procedures. SCNT was performed as described previously in the laboratory.^[^ ^18^, ^31^, ^33^ ^]^ Following SCNT, high‐quality embryos were selected for embryo transfer. Embryos at the one to two‐cell stage, cultured for 20–24 h post‐SCNT, were transferred into the oviducts of synchronized recipient goats on day 1 of estrus, with day 0 designated as the day of estrus. Each recipient received 19–28 embryos. Pregnancy status was evaluated via ultrasonography 60 d after embryo transfer to determine the success of the recipient's pregnancy.

### Establishment of Goats Mastitis Model

*E.coli* (25 922) was maintained in the laboratory following established protocols.^[^ ^34^ ^]^ Twelve primiparous Saanen goats with the same genetic background, close in body weights and daily milk yields were grouped to screen for optimal concentrations of *E. coli* causing mastitis. Prior to initiating the infection experiment, goats were observed in a thoroughly clean site for 14 d. Mastitis was tested daily for 7 d before *E. coli* infection, and all dairy goats that tested negative for bacteriology had a milk SCC of less than 5 × 10^5^ cells mL^−1^. The experiment consisted of a control group (both mammary gland were untreated, receiving the same dose of sterile PBS, n = 3), *E. coli* 1 group (concentration of 6 × 10^2^ CFU mL^−1^, both mammary gland were treated, n = 3), *E. coli* 2 group (6 × 10^4^ CFU mL^−1^, both mammary gland were treated, n = 3), and *E. coli* 3 group (6 × 10^6^ CFU mL^−1^, both mammary gland were treated, n = 3), which were infected with *E. coli* for 7 consecutive days. The SCC, IL‐6, IL‐1β, and TNFα in the milk were detected on the 1st, 3rd, 5th, 7th, and 9th days post‐infection, with infections conducted consistently at the same time each day, and the degree of mastitis was determined based on SCC levels in the milk samples. *E. coli* was infused into the mammary glands of the experimental goats via the papillary canal. Before *E. coli* infusion, washed with warm water, and the nipple was sterilized with 75% alcohol. Then, a sterile syringe was used to administer the *E. coli* suspension into the udder through the lactation needle. Following injection, the lactation needle was removed, and gentle massage of the mammary gland ensured uniform dispersion of the bacterial solution. Throughout the experiment, the goats were permitted normal movement within their pen and were fed according to their regular diet, ensuring conditions consistent with their typical living environment. The final concentration of *E. coli* used was determined to be 6 × 10^6^ CFU mL^−1^.

WTD goats, sharing identical genetic traits, were chosen as the control group. GED goats (n = 3) and WTD goats (n = 3) were subjected to natural mating and parturition until they reached the lactation stage. One mammary gland of each GED goat was surgically (minimally invasive) sampled to isolate gene‐edited GMEC for subsequent verification tests. The other mammary gland was utilized to evaluate bacterial infiltration, following the same procedure as described earlier. Tissue samples from the infected mammary glands were collected on the 9th day post‐infection, following a 7 d infection period.

### Off‐target Analysis

Off‐target assays were performed according to methods previously reported.^[^ ^4^, ^35^ ^]^ To evaluate the specificity of TnpB, the CRISPR RGEN Tools (Cas‐OFFinder, http://www.rgenome.net/cas‐offinder/) was used to predict the potential off‐target sites. Search queries were 20‐nt on‐target TnpB spacer sequences. The PAM of research was set to TTGAT, and the mismatches were set to 5. All other parameters were left as default. Based on the prediction results, 7 sites with high off‐target probability were selected to design primers using the Primer Premier 5 (PREMIER Biosoft, Canada). The predicted potential off‐target sequences were amplified and Sanger sequencing to evaluate the specificity of TnpB. All predicted potential off‐target sites and primers used in off‐target analysis were provided in Table S3 (Supporting Information).

### RT‐qPCR

RT‐qPCR was performed as previously described.^[^ ^25b^ ^]^ Briefly, cells from various treatment groups were collected in Trizol reagent (15596026CN, Thermo Fisher Scientific inc, USA) for RNA extraction. Subsequently, 2 µg of isolated RNA was reverse transcribed into cDNA using the reverse transcription kit (Takara Bio Inc., China), following the manufacturer's instructions. Each synthesized cDNA sample was diluted to 100 µL with RNase‐free water for use in subsequent experiments. RT‐qPCR was used to determine gene expressions on QuantStudio design and analysis software (Applied Biosystems, USA). The reaction mixture (10 µL) consists of 5 µL FastStart Universal SYBR Master (ROX) (Roche, USA), with 0.3 µL each of the forward and reverse primers and 3.4 µL RNase‐free water. Table S1 (Supporting Information) below lists the detailed information of the primers.

### Western Blotting

Western blotting was performed as previously described.^[^ ^25b^ ^]^ Briefly, cells from different treatment groups were collected, and they were lysed in RIPA buffer (Santa Cruz, USA) containing protease inhibitor cocktail (Santa Cruz, USA). Protein concentrations of the extracts were measured by the enhanced BCA protein assay kit (P0009, Beyotime, China) according to the manufacturer's instructions. Then separated by 10% and 12% SDS‐PAGE. The proteins were transferred onto the PVDF membrane (Roche, USA), which was then blocked in a 5% skim milk blocking solution for 2 h at room temperature. Following blocking, the membranes were incubated with primary antibodies overnight at 4 °C. They were washed three times with TBST and then incubated at room temperature for 2 h with a secondary antibody. Following this, the membranes were washed three times with TBST and then exposed to an ECL color‐developing solution (32 106, Thermo Fisher Scientific Inc, USA). The immunoreactive bands were detected using the ECL Western blotting detection system (ChemiDoc image analysis system, BIO‐RAD, USA). Band densitometry was quantified using Image J software (NIH, Bethesda, MD). Table S4 (Supporting Information) below lists the detailed information of the antibody.

### IF Analysis of Cells and Tissues

Table S4 (Supporting Information) below lists the detailed information of the antibody. IF was performed as previously described.^[^ ^25b^ ^]^ Briefly, the primary antibody (diluted 1:100 in 1% BSA) was applied and allowed to incubate with the samples overnight at 4 °C. Following 3 washes with PBS for 10 min each, the secondary antibody (diluted 1:500) was applied and incubated for 1 h at room temperature in the dark. Subsequent to 3 additional washes with 1 × PBS, the nuclei were stained with DAPI (C1002, Beyotime, China) for 2 min. Finally, confocal microscopy (Nikon, Tokyo, Japan) was used to image the cells.

IF assay of mammary gland tissue was performed according to previously reported methods,^[^ ^36^ ^]^ sent to Shaanxi Yike Biotechnology Service Co., Ltd. (Shaanxi, China) for experiments, paraffin‐embedded goats mammary gland sections were deparaffinized for subsequent antigen retrieval, and antigen repair solution for antigen repair. Antibodies were incubated according to strict instructions, and images were captured using a fluorescence microscope (Nikon, Tokyo, Japan).

### ELISA

The dairy goats LYZ ELISA kit (JL50435) was purchased from Jianglai Biological (Shanghai, China), while dairy goats IL‐6 ELISA kit (MM‐35226O2), dairy goats TNFα ELISA kit (MM‐0096O2), and dairy goats IL‐1β ELISA kit (MM‐1751O2) were obtained from Meimian Industrial (Jiangsu, China). The operation and data analysis were performed in strict accordance with the manufacturer's instructions.

### Flow Cytometry Analysis

Cells were seeded into 6‐well plates at a density of 1 × 10^6^ cells mL^−1^. Following LPS treatments, the cells were harvested, washed with cold PBS, and suspended in 500 µL of staining solution (C1075S, Beyotime, China), then incubated at 37 °C for 20 min in the dark. Flow cytometry was utilized to analyze the cell phenotype using FACSAria III (Becton Dickinson, New Jersey, USA) within 1 h.

### Microscopy Imaging for Cell Death

Cells were inoculated in a 24‐well plate at a density of 5 × 10^5^ cells mL^−1^. Following LPS treatments, cells were stained with oxazole yellow/propidium iodide staining fluid (C1075S, Beyotime, China) as per the manufacturer's instructions. The apoptotic cells showed green fluorescence, while the necrotic cells expressed red fluorescence under fluorescence microscopy (Nikon, Kawasaki, Japan).

### HE Staining

The collected mammary gland tissues of goats were fixed in 4% formaldehyde solution and sent to Shaanxi Yike Biotechnology Service Co., Ltd.(Shaanxi, China) for experiments. The tissues were then embedded in paraffin and cut into 5‐µm thick sections using a microtome (RM2245, Wetzlar, Germany). The sections were stained with HE, and histological analysis was performed using an optical microscope (Olympus, Tokyo, Japan).^[^ ^37^ ^]^

### GMEC Organoids

Supplementary Table S5 below lists the detailed information of the organoid medium composition. GMEC organoid establishment methods was performed as previously described.^[^ ^38^ ^]^ Briefly, dairy goats mammary gland tissue was obtained using surgical methods. The tissue was then placed in a 15 mL centrifuge tube and digested with collagenase in an incubator at 37 °C for 5 h with occasional inversion every 30 min until no visible particles remained. Following digestion termination, the cell suspension was filtered and counted using a 70 µm cell sieve. The cell concentration was adjusted to ≈1 × 10^5^ cells mL^−1^, and the cells were resuspended in matrigel before inoculation into 24‐well plates. Once the matrigel solidified, 700 µL of organoid medium was added to each well, and the plates were incubated at 37 °C in a 5% CO_2_. Gene‐edited GMEC organoids were obtained consistent with the methods described above.

### IF Analysis of Organoids

Briefly, after discarding the medium, 1 mL of pre‐cooled cell recovery solution was added to each well, and the matrigel was disrupted by gentle pipetting. The contents were transferred to new 1.5 mL centrifuge tubes and incubated at 4 °C for 30 min. Following incubation, centrifugation was performed at 4 °C and 400 × g for 5 min, and the supernatant was discarded. Each well was then filled with 1 mL of 4% paraformaldehyde and transferred to a 24‐well plate containing cell climbing sheets, where they were left overnight at 4 °C for fixation. Subsequently, 200 µL of Triton X‐100 was added to each well for 20 min. Next, 500 µL of blocking solution was added to each well for overnight blocking or an appropriate extension of time. Primary antibody was diluted at a ratio of 1:100, and 200 µL of the diluted primary antibody was added to each well for incubation at 4 °C for 1 d. The secondary antibody was diluted at a ratio of 1:500, and 200 µL of the diluted secondary antibody was added to each well for incubation at 4 °C for 1 d. Finally, 200 µL of DAPI was added to each well and stained for 3 min. After discarding the DAPI (C1002, Beyotime, China) solution, PBS was used to rinse the wells before observing under fluorescence microscopy (Nikon, Kawasaki, Japan) (Nikon, Tokyo, Japan). Table S5 (Supporting Information) below lists the detailed information of the antibody.

### Computational Processing and Bioinformatics of RNA‐seq

After waiting for the completion of cell treatment, samples are collected in strict accordance with the requirements and sent to Novogene sequencing company (Novogene Co., Ltd. Beijing, China) for RNA‐seq. The transcriptome data were deposited in the National Center for Biotechnology Information (https://www.ncbi.nlm.nih.gov/sra/), BioProject ID was PRJNA1090220. The reference genome for goats was downloaded from Ensembl website (https://asia.ensembl.org/index.html). Read count matrices were obtained using Feature Counts. DEGs were accessed using DESeq2, with a false discovery rate less than 0.05 and |log2Fold Change| >1. Both GO and KEGG functional enrichment analysis or visualization were finished by R package ClusterProfiler or ClueGO, a plug‐in of Cytoscape. For the heatmap analysis, clustering analysis was performed through the Hlipot website (https://hiplot.com.cn) with the clustering method ward.D2.

### Statistical Analysis

The experimental data were analyzed by SPSS 20 software. All data were displayed as the mean ± SEM and from at least three independent experiments. Statistical analysis between two groups was performed using unpaired t‐test, and one‐way ANOVA were employed for multiple groups. Statistical significance was taken as a *P* value less than 0.05. *: indicates significant difference (*P* < 0.05), **: indicates that the difference is highly significant (*P* < 0.01) and ns: indicates no significant difference (*P* > 0.05). All data were analyzed and graphed using GraphPad Prism 8.0 software.

## Conflict of Interest

The authors declare no conflict of interest.

## Author Contributions

R.F., J.Z., Q.Z., and Z.Z. contributed equally to this work. R.F. performed investigation, data curation, methodology and wrote the original draft; J.Z., Q.Z., and Z.Z. performed investigation and validation; J.S., C.L. provided software; J.Z., X.Z., X.M., X.Y.M., L.G., Y.L., X.Y., and F.W. performed formal analysis; H.L., X.C., Y.D., and G.W. performed visualization and resources; Y.Z. performed methodology; X.L. and J.L. performed conceptualization, visualization, supervision and wrote‐reviewed and edited.

## Supporting information

Supporting Information

## Data Availability

The data that support the findings of this study are available from the corresponding author upon reasonable request.
