# Supplementary material for: Generation of Anti‐Mastitis Gene‐Edited Dairy Goats with Enhancing Lysozyme Expression by Inflammatory Regulatory Sequence using ISDra2‐TnpB System
Source: Adv Sci (Weinh). 2024 Aug 5;11(38):2404408. doi: 10.1002/advs.202404408 (PMC11481229; doi:10.1002/advs.202404408)
Supplement: Supplementary file 1 — Supporting Information [file ADVS-11-2404408-s001.docx]

Supporting Information for

**Generation of Anti-mastitis Gene-edited Dairy Goats with Enhancing Lysozyme Expression by Inflammatory Regulatory Sequence using ISDra2-TnpB System**

*Rui Feng^1^, Jianglin Zhao^1^, Qian Zhang^1^, Zhenliang Zhu^1^, Junyu Zhang, Chengyuan Liu, Xiaoman Zheng, Fan Wang, Jie Su, Xianghai Ma, Xiaoyu Mi, Lin Guo, Xiaoxue Yan, Yayi Liu, Huijia Li, Xu Chen, Yi Deng, Guoyan Wang, Yong Zhang, Xu Liu* and Jun Liu**

**This word file includes:**

Figure S1 to Figure S20

Table S1 to Table S5


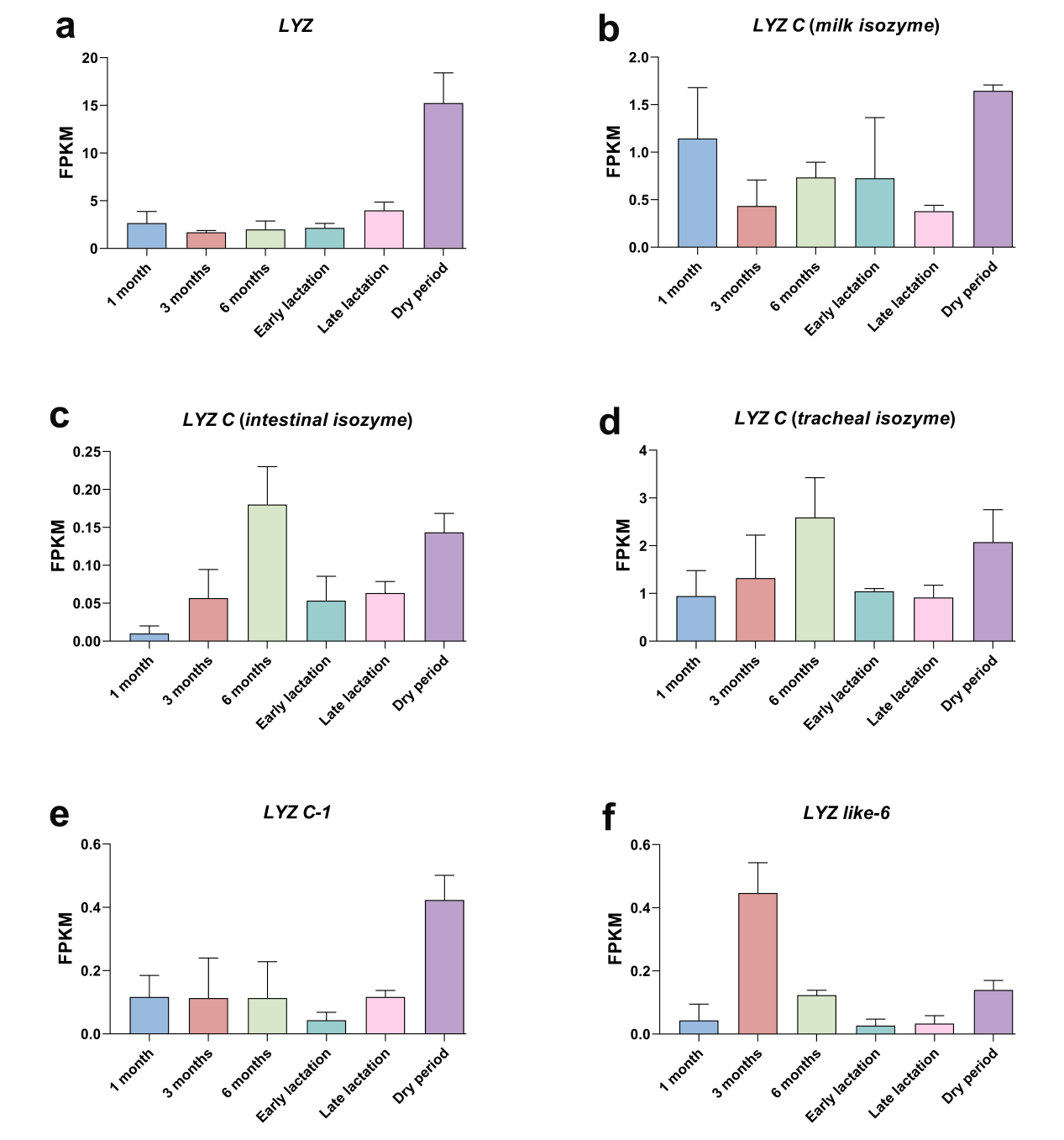
**Figure S1. Analysis of different types of *LYZ* expression in the mammary gland of dairy goat.** (a) *LYZ*, NCBI ID is 100860864. (b) *LYZ C* (milk isozyme), NCBI gene ID is 102171764. (c) *LYZ C* (intestinal isozyme), NCBI gene ID is 108636006. (d) *LYZ C* (tracheal isozyme), NCBI gene ID is 108633237. (e) *LYZ C-1*, NCBI gene ID is 102172037. (f) *LYZ like-6*, NCBI gene ID is 102190166.

**
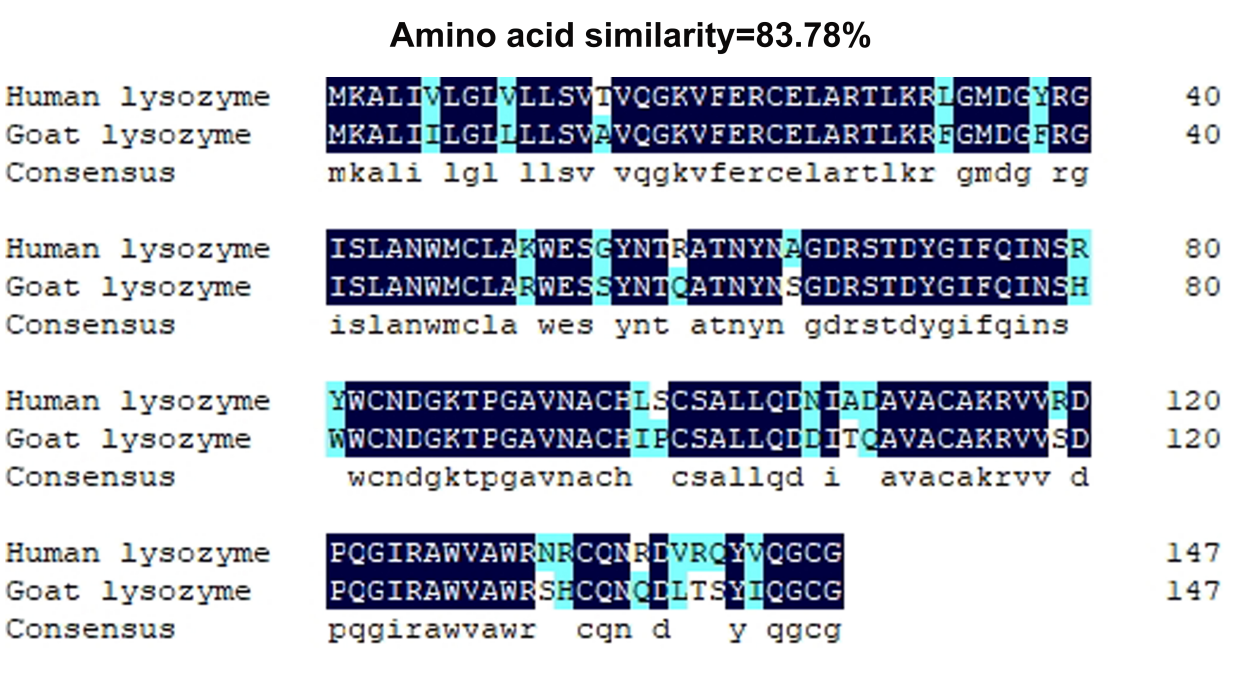
Figure S2. Comparison of amino acid homology between human LYZ and goats LYZ.**

**
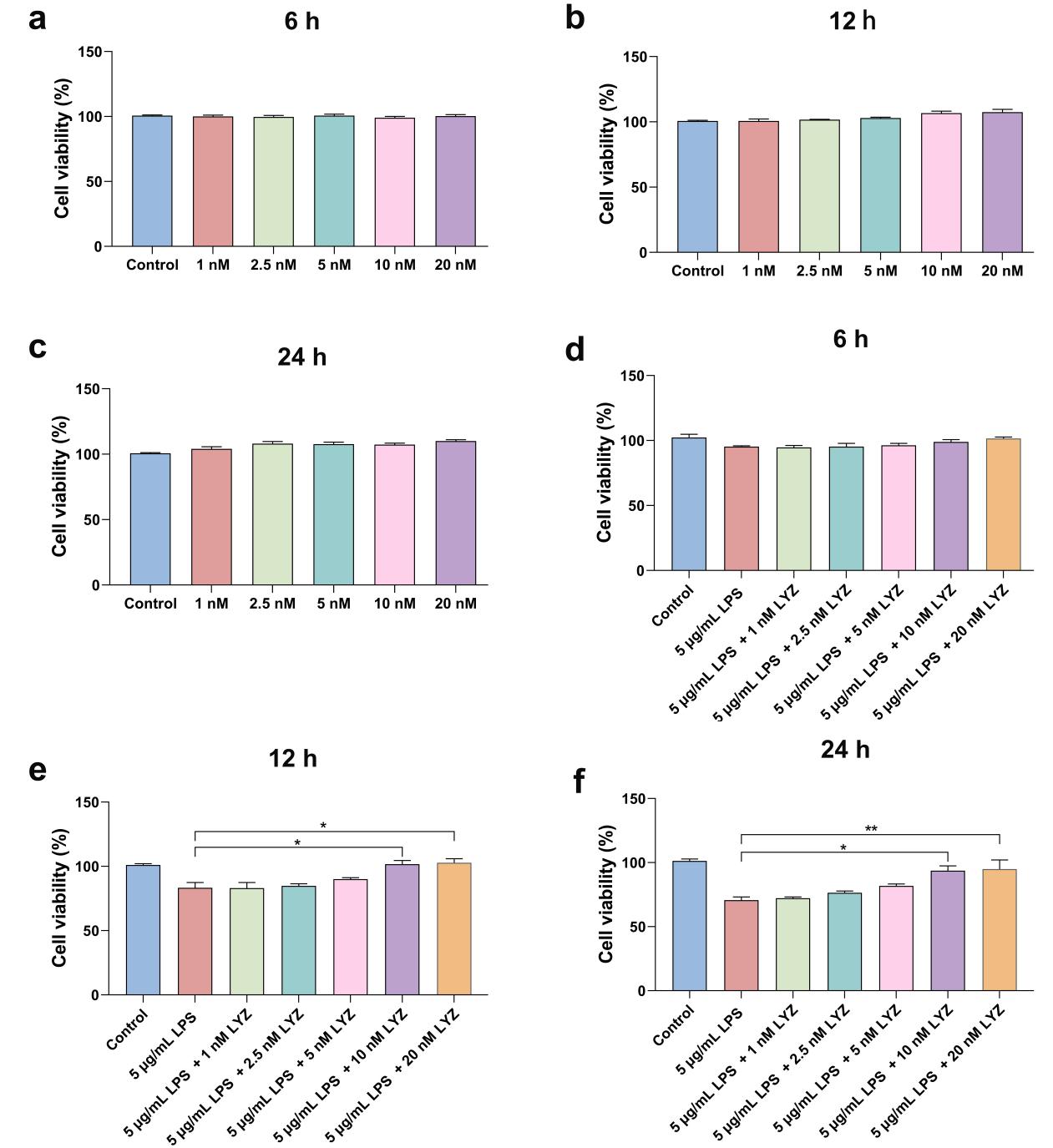
Figure S3****. Effect of dairy** **goat LYZ recombinant protein on GEMC viability.** (a-c) GMEC was treated with different concentrations of LYZ recombinant proteins for 6, 12 and 24 h, and cell viability was detected. (d-f) GMEC was treated with 5 μg mL^-1^ LPS for 12 h, and then treated with different concentrations of LYZ recombinant protein for 6, 12 and 24 h, and cell viability was detected. Values are expressed as mean ± SEM (n=3 per group) by one-way ANOVA. *: indicates significant difference (*P* < 0.05), **: indicates that the difference is highly significant (*P* < 0.01) and ns: indicates no significant difference (*P* > 0.05).

**
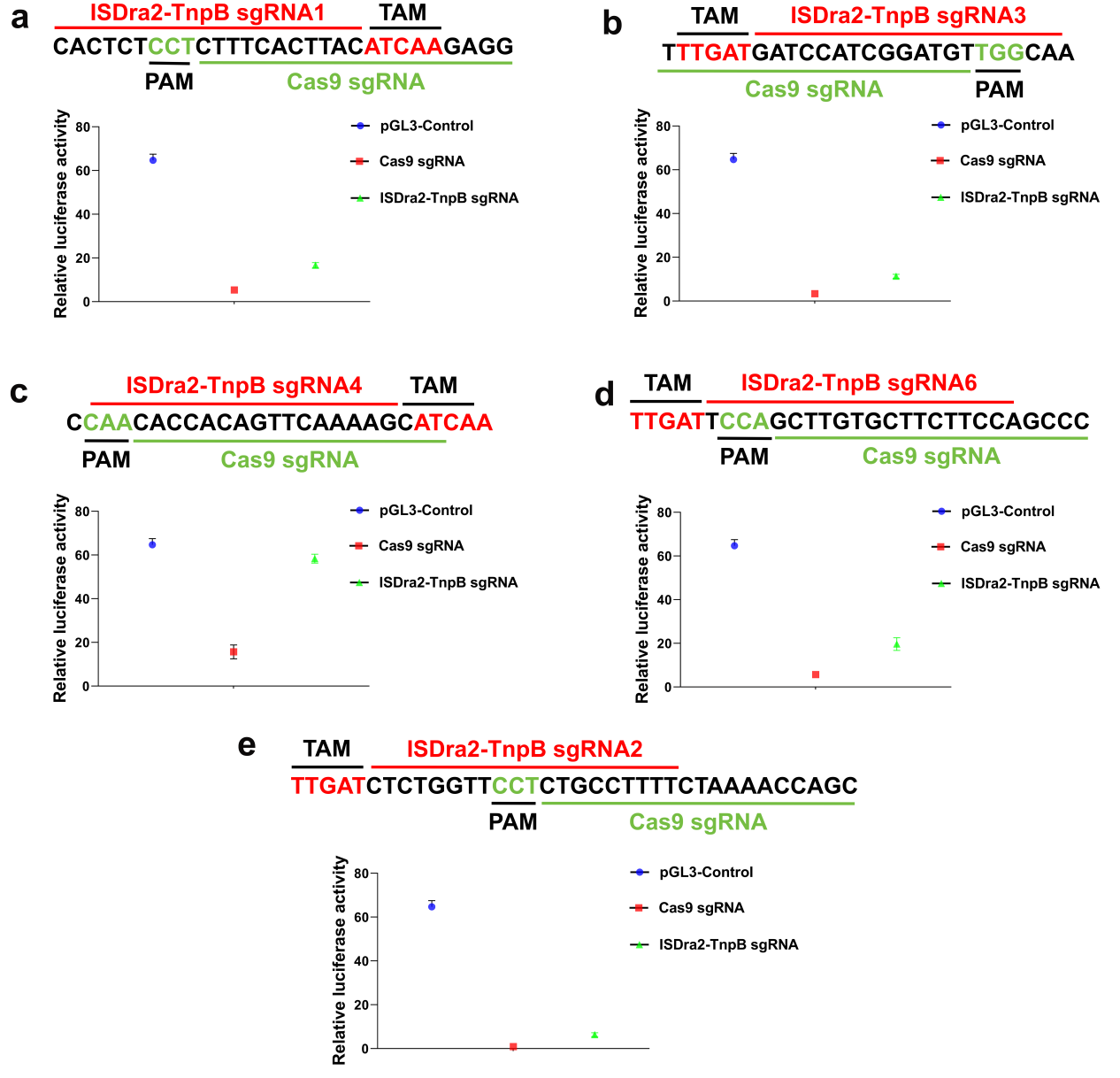
Figure S4****. Comparison of ISDra2-TnpB and CRISPR/Cas9 cleavage activity at the same sgRNAs site using SSA method.** (a-e) The cleavage activities of ISDra2-TnpB and CRISPR/Cas9 at the sgRNA1, sgRNA2, sgRNA3, sgRNA4 and sgRNA6 site, respectively, were analyzed using the SSA method (n=3 per group).


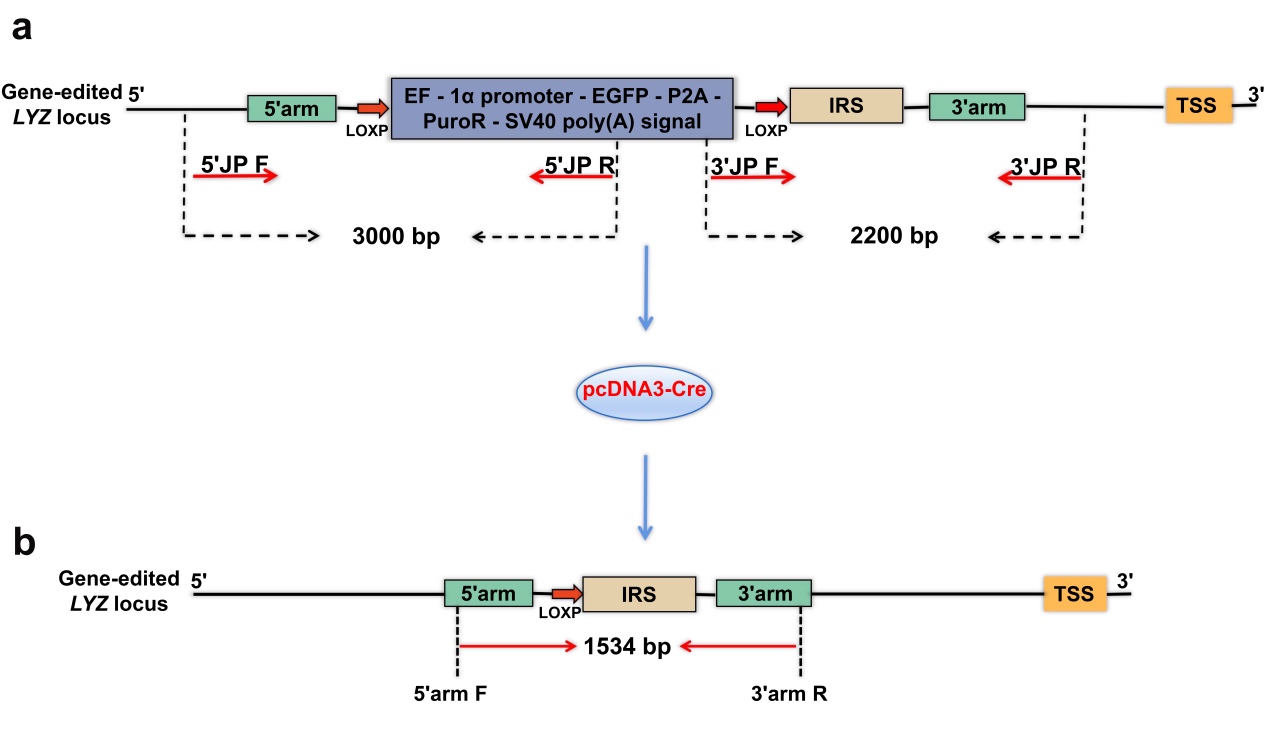
**Figure S5. Schematic overview of the screening of the individual colonies.** (a) Schematic overview of the screening of the individual colonies screening with green fluorescent labeling. 5ʹJP F and 3ʹJP R were the primers for the regions outside the homologous arms, and 5ʹJP R and 3ʹJP F were the primers for the donor vector region. The products were 3000bp and 2200bp, respectively. (b) Schematic overview of the screening of the individual colonies screening after treatment with the Cre/LOXP system. 5'am F and 3'am R were the primers for the regions inside the homologous arms. The products were 1534bp.

**
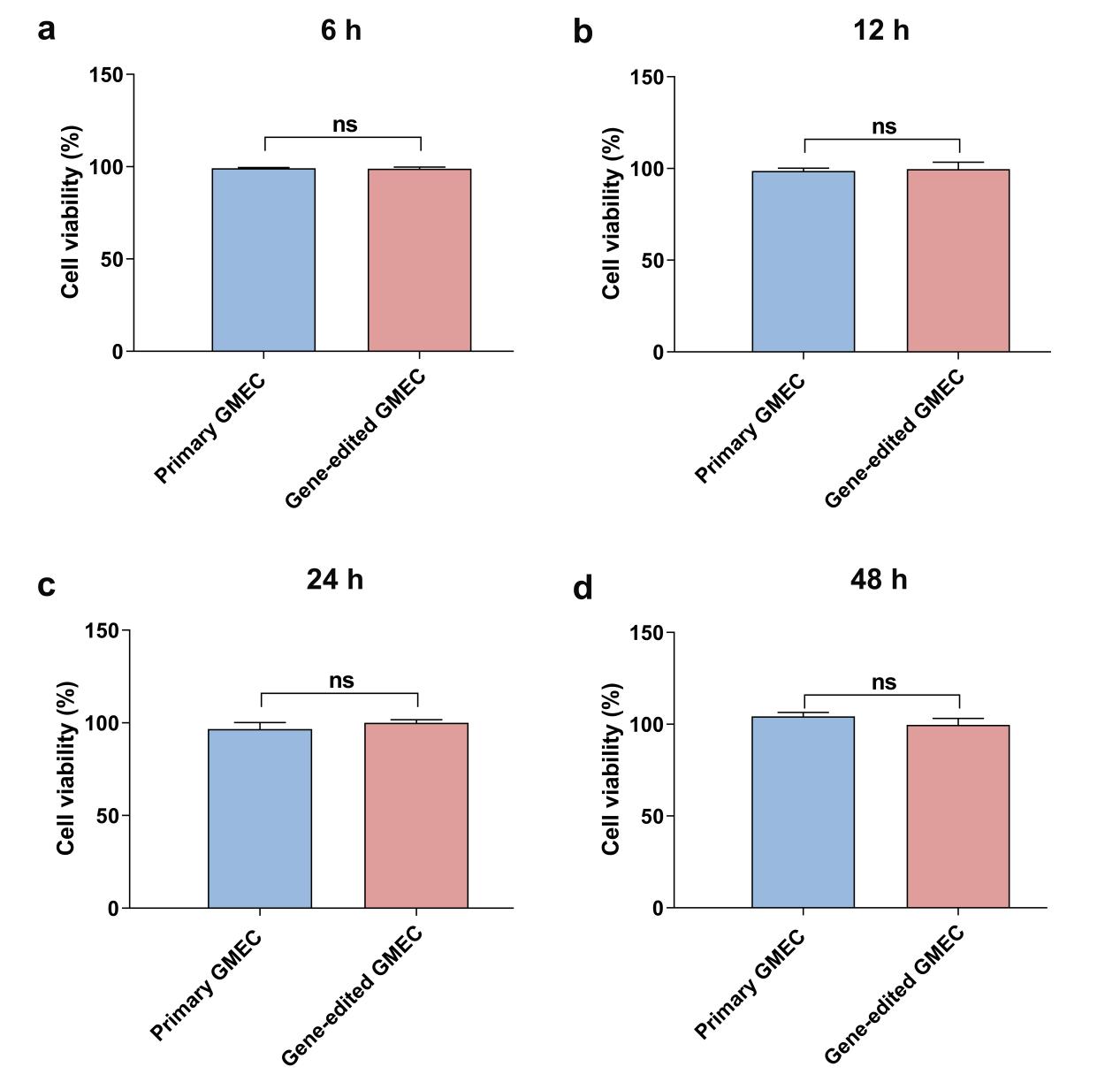
**

**Figure S6****. Viability analysis of primary GMEC and gene-edited GMEC.** (a-d) Cultured primary GMEC and gene-edited GMEC for 6, 12, 24 and 48 h to detect cell viability. Values are expressed as mean ± SEM (n=3 per group) by unpaired t-test. *: indicates significant difference (*P* < 0.05), **: indicates that the difference is highly significant (*P* < 0.01) and ns: indicates no significant difference (*P* > 0.05).


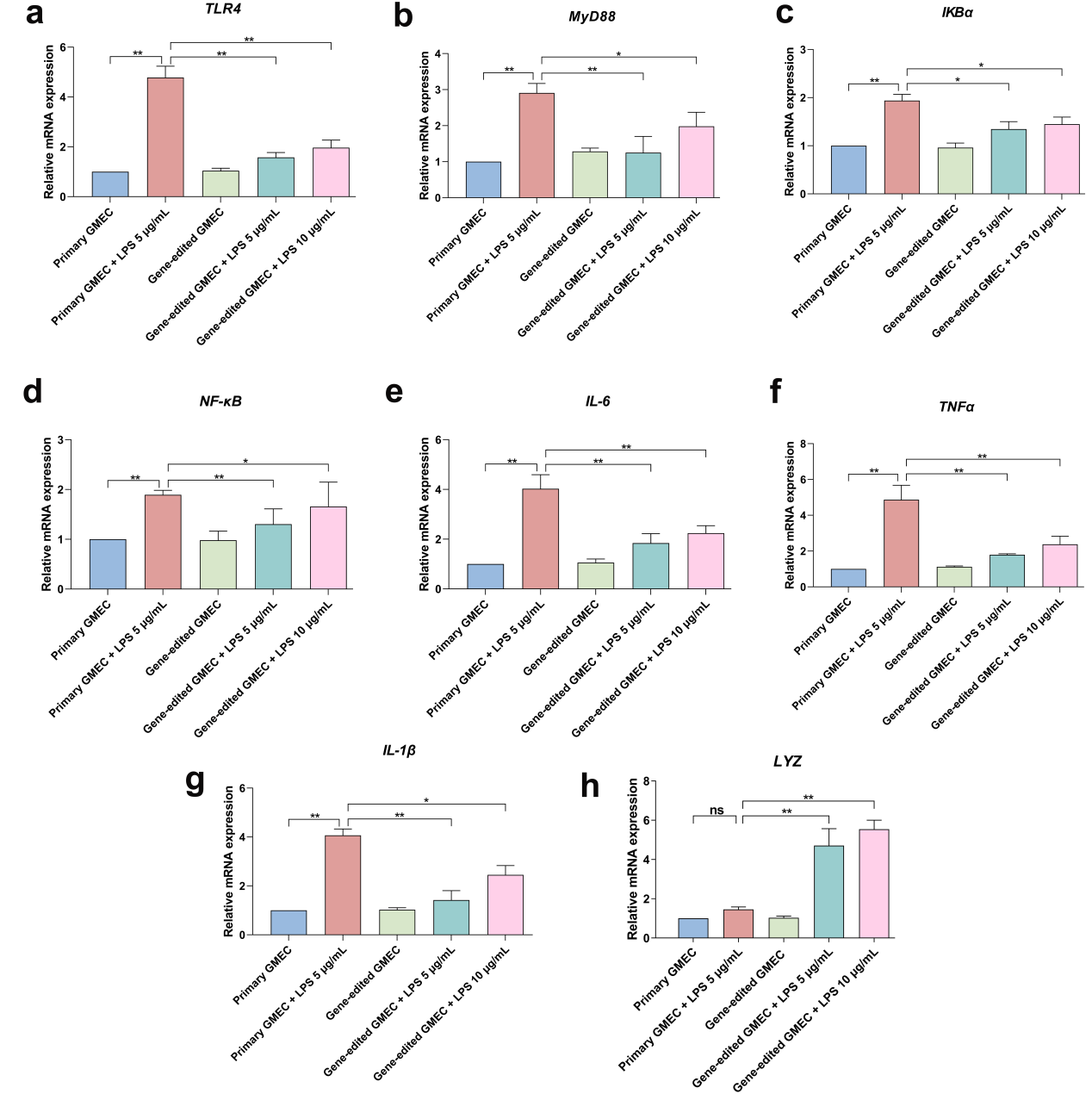
**Figure S7. Analysis of mRNA expression of inflammation-related genes in gene-edited GMEC under inflammatory conditions.** (a-h) Analysis of mRNA expression of *TLR4*, *MyD88*, *IKBα*, *NF-κB*, *IL-6*, *TNFα*, *IL-1β* and *LYZ*. Values are expressed as mean ± SEM (n=3 per group) by one-way ANOVA. *: indicates significant difference (*P* < 0.05), **: indicates that the difference is highly significant (*P* < 0.01) and ns: indicates no significant difference (*P* > 0.05).


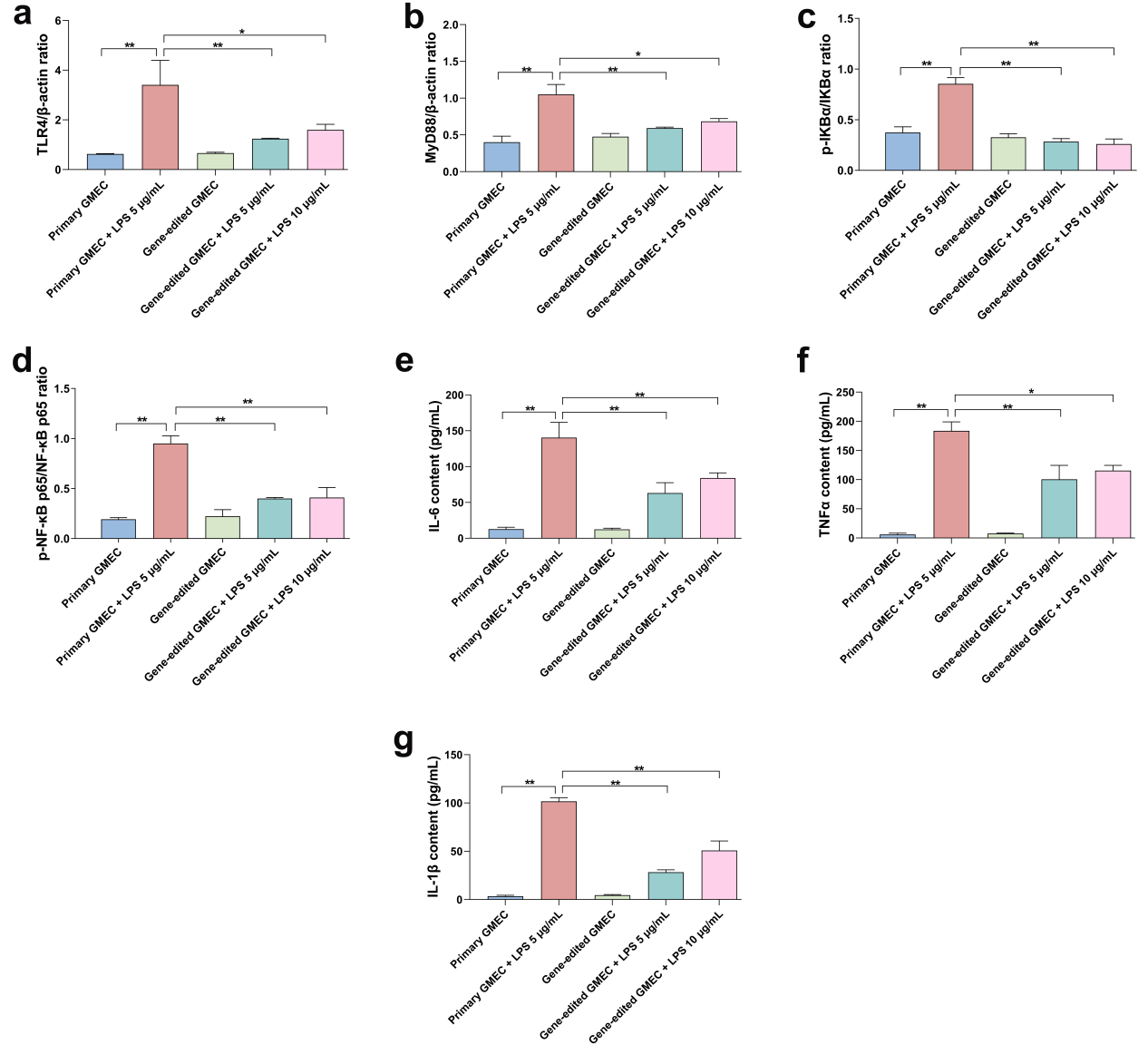
**Figure S8. Analysis of inflammation-related protein expression in gene-edited GMEC under inflammatory conditions.** (a-b) The protein ratio of TLR4, MyD88 and β-actin. (c) The protein ratio of p-IKBα and IKBα. (d) The protein ratio of p-NF-κB p65 and NF-κB p65. (e-g) Analysis of IL-6, TNFα, and IL-1β protein expression. Values are expressed as mean ± SEM (n=3 per group) by one-way ANOVA. *: indicates significant difference (*P* < 0.05), **: indicates that the difference is highly significant (*P* < 0.01) and ns: indicates no significant difference (*P* > 0.05).

**
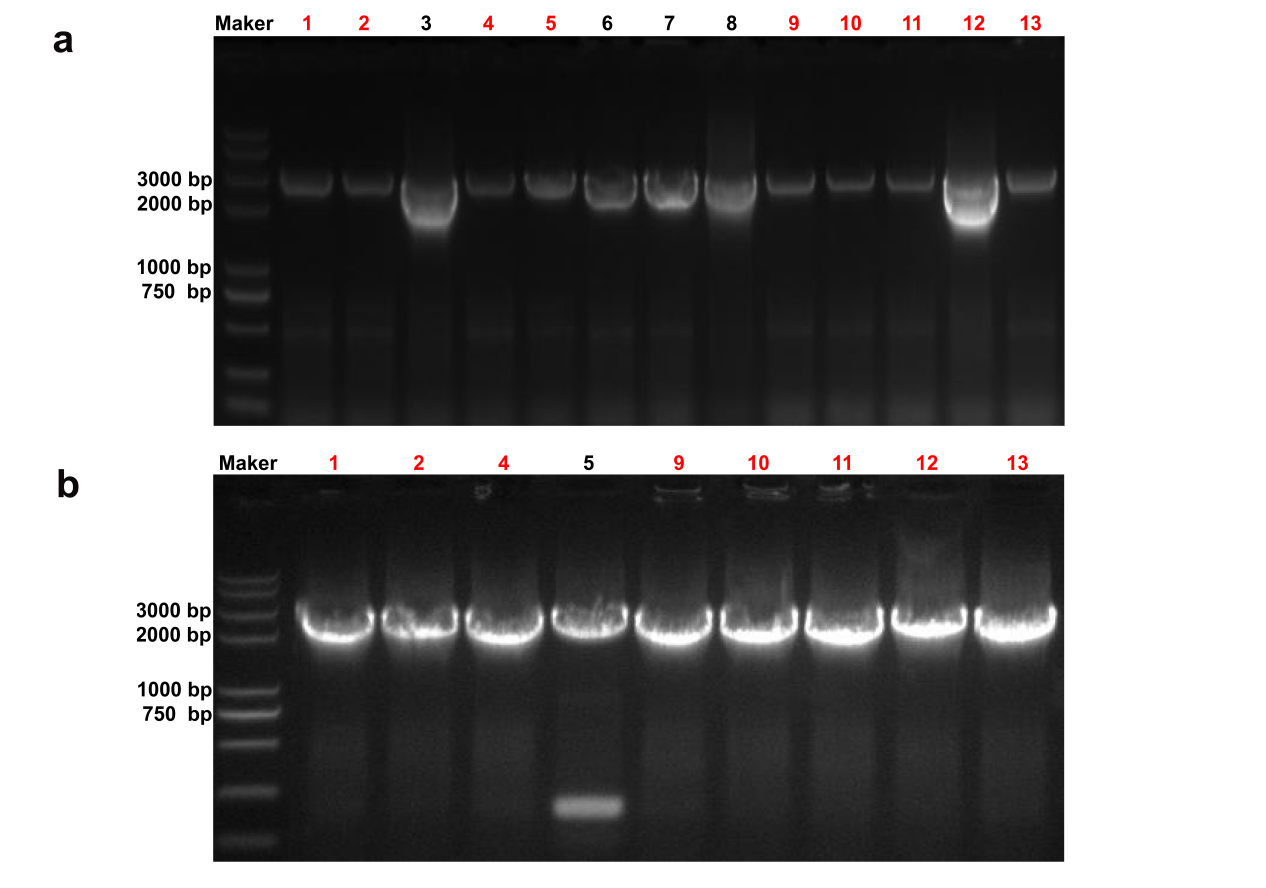
Figure S9. Junction PCR identification.** (a-b) Drug-resistant colonies 5ʹ-junction PCR and 3ʹ-junction PCR results with green fluorescent screening markers. The products were 3000bp and 2200bp.


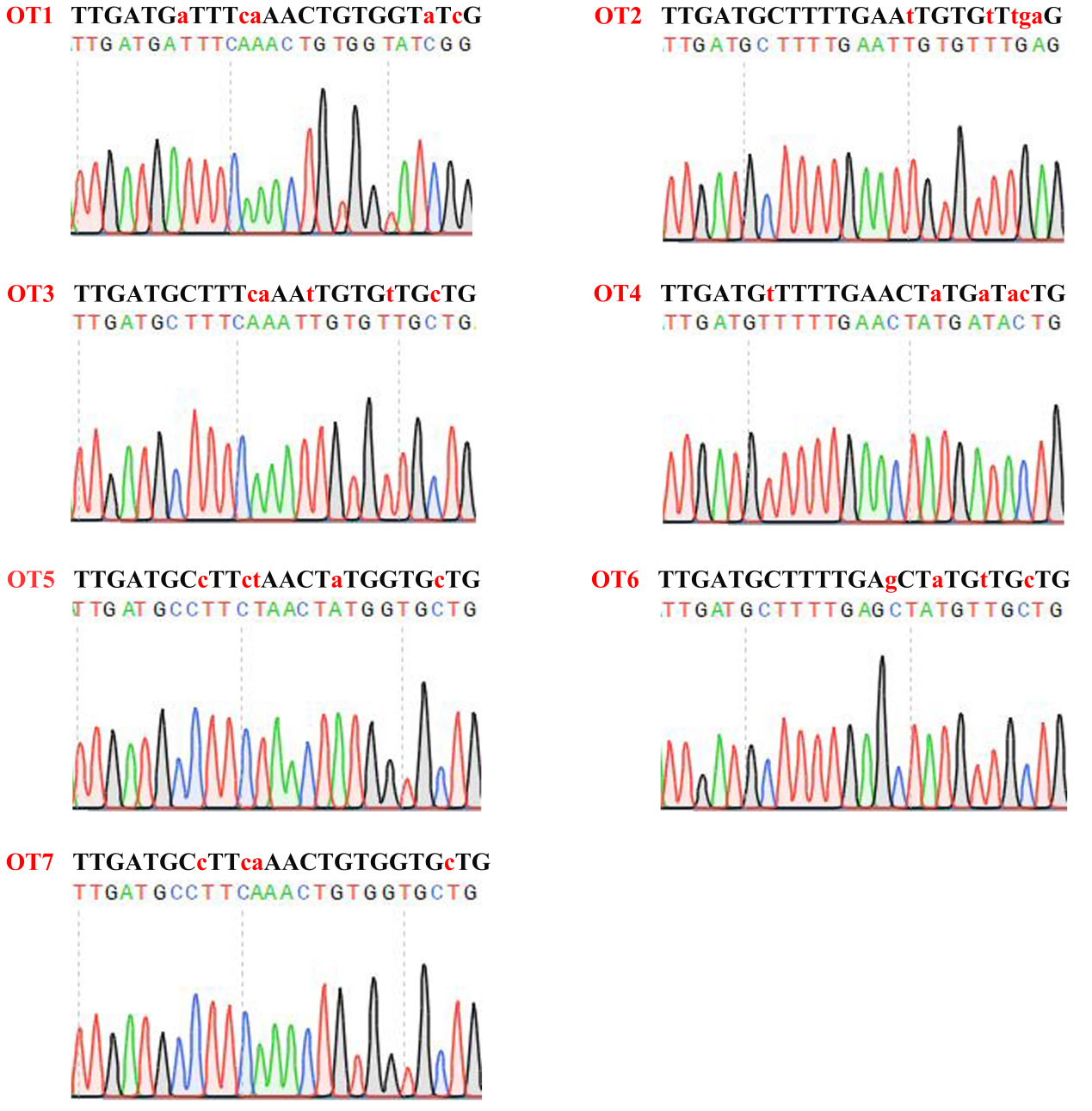
**Figure S10. Off-target analysis of GED goats.** (a-h) Seven major sgRNA4 potential off-target sites from all GED goat genomes were cloned and validated using sanger sequencing.


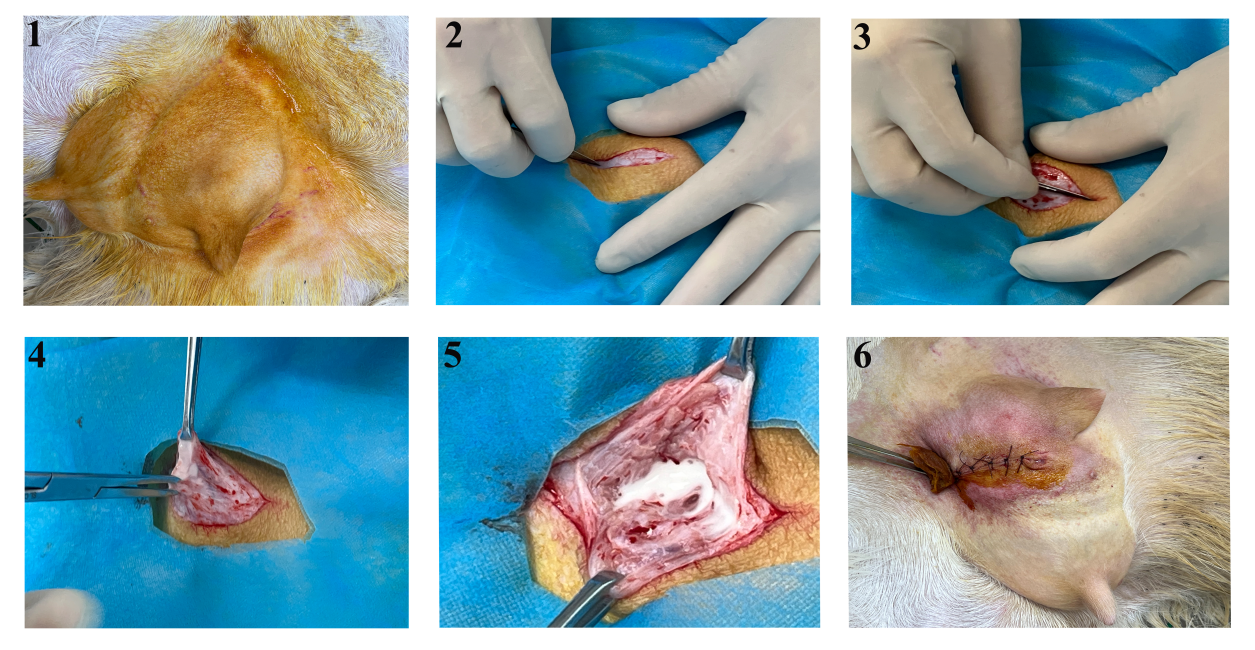


**Figure S11****. The process of obtaining mammary gland tissue surgically.**


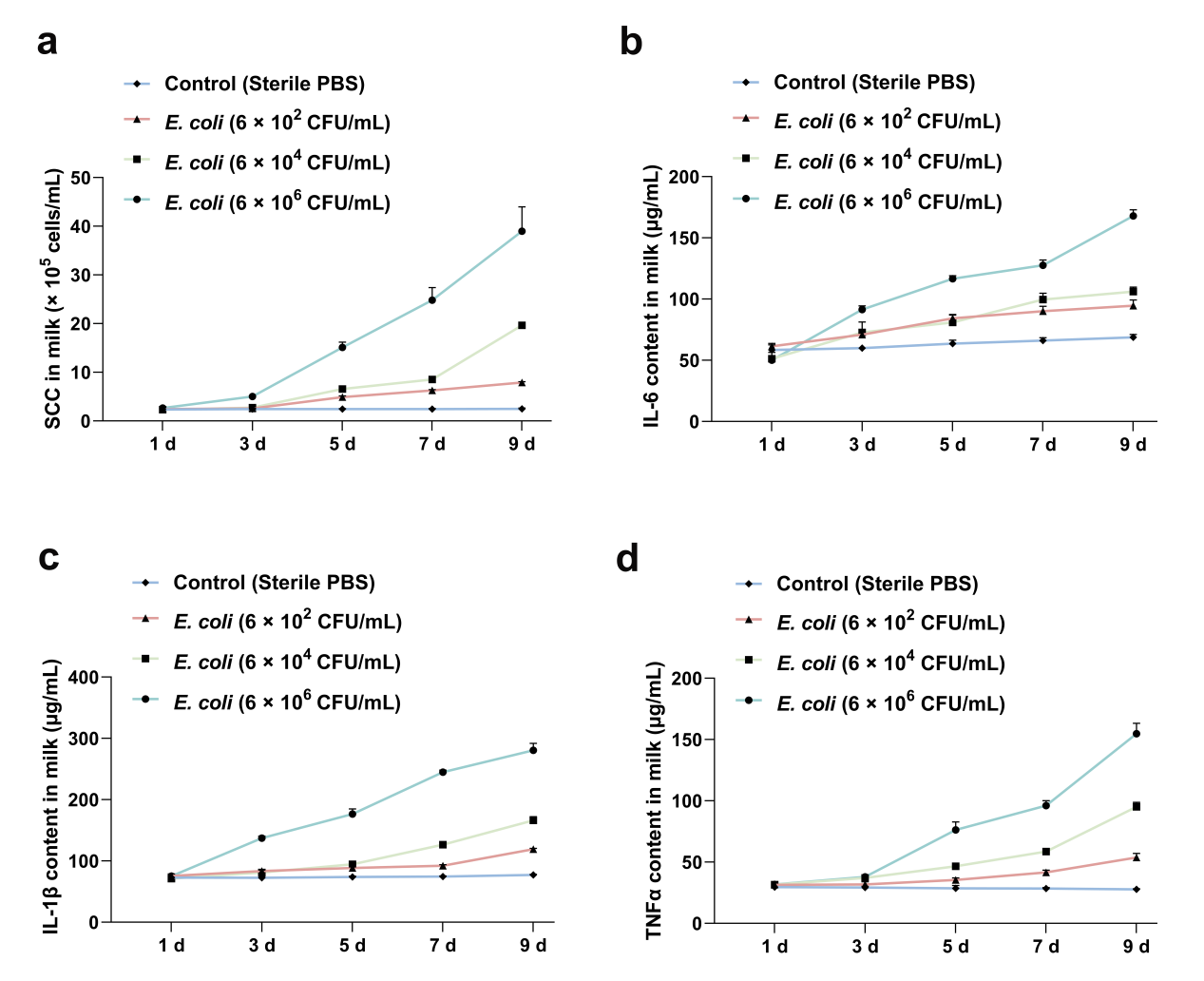


**Figure S12. Analysis of SCC, IL-6, IL-1β, and TNFα content in milk.** WTD dairy goats were treated with sterile PBS and *E. coli*, respectively. (a) Analysis of SCC in milk. (b-d) Analysis of IL-6, IL-1β, and TNFα content in milk. Values are expressed as mean ± SEM (n=3 per group) by one-way ANOVA. *: indicates significant difference (*P* < 0.05), **: indicates that the difference is highly significant (*P* < 0.01) and ns: indicates no significant difference (*P* > 0.05).


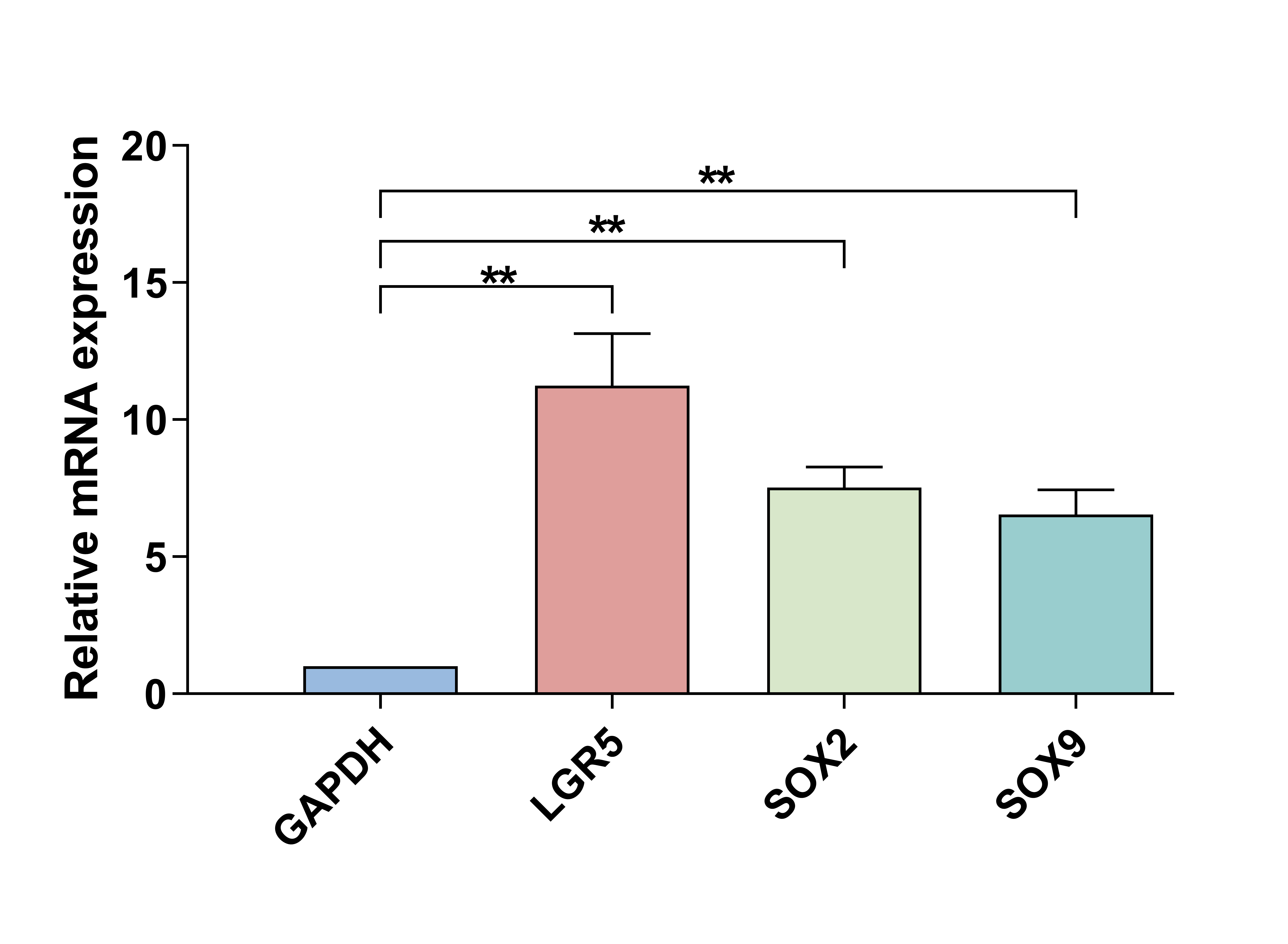
**Figure S13. analysis the mRNA expression of *LGR5*, *SOX2* and *SOX9*** **in GMEC organoids.** Analysis of *LGR5*, *SOX2* and *SOX9* mRNA expression. Values are expressed as mean ± SEM (n=3 per group) by one-way ANOVA. *: indicates significant difference (*P* < 0.05), **: indicates that the difference is highly significant (*P* < 0.01) and ns: indicates no significant difference (*P* > 0.05).


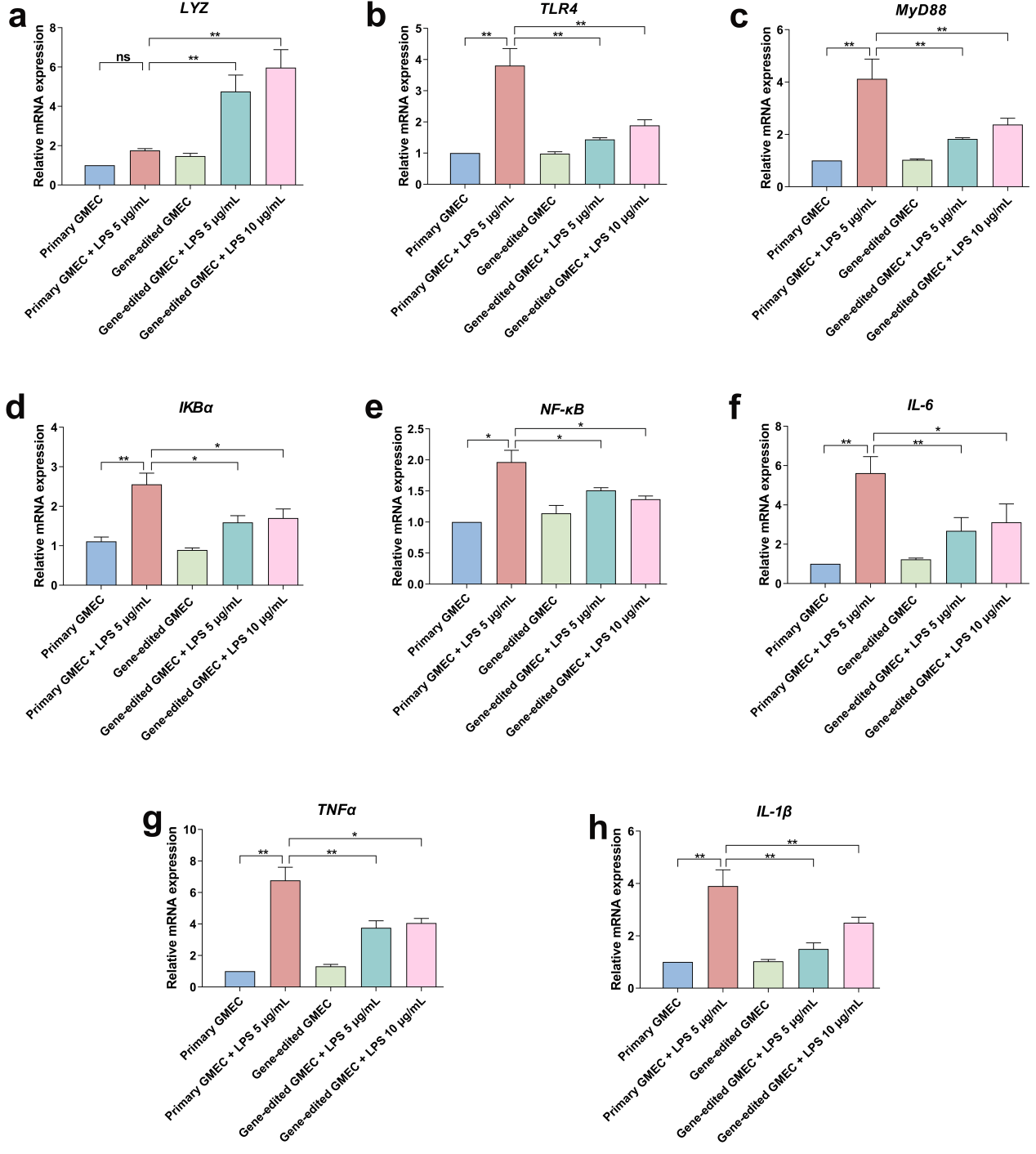
**Figure S14. Analysis of mRNA expression of inflammation-related genes in GED goats mammary epithelial cells under inflammatory conditions.** (a-h) Analysis of *LYZ*, *TLR4*, *MyD88*, *IKBα*, *NF-κB*, *IL-6*, *TNFα* and *IL-1β* mRNA expression. Values are expressed as mean ± SEM (n=3 per group) by one-way ANOVA. *: indicates significant difference (*P* < 0.05), **: indicates that the difference is highly significant (*P* < 0.01) and ns: indicates no significant difference (*P* > 0.05).


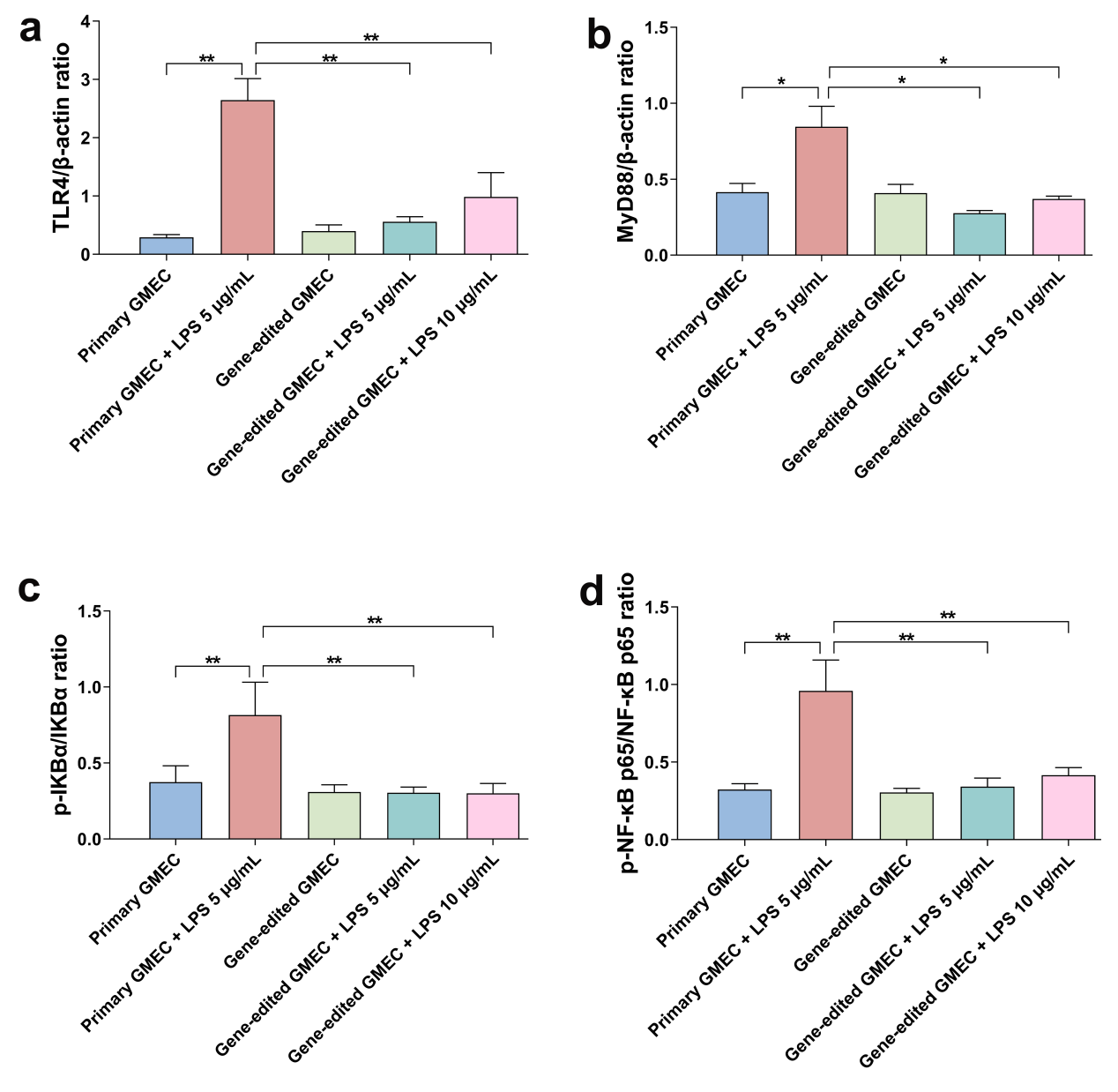
**Figure S15. Analysis of inflammation-related protein expression in GED goats mammary epithelial cells under inflammatory conditions.** (a) The protein ratio of TLR4, MyD88 and β-actin. (c) The protein ratio of p-IKBα and IKBα. (d) The protein ratio of p-NF-κB p65 and NF-κB p65. Values are expressed as mean ± SEM (n=3 per group) by one-way ANOVA. *: indicates significant difference (*P* < 0.05), **: indicates that the difference is highly significant (*P* < 0.01) and ns: indicates no significant difference (*P* > 0.05).

**
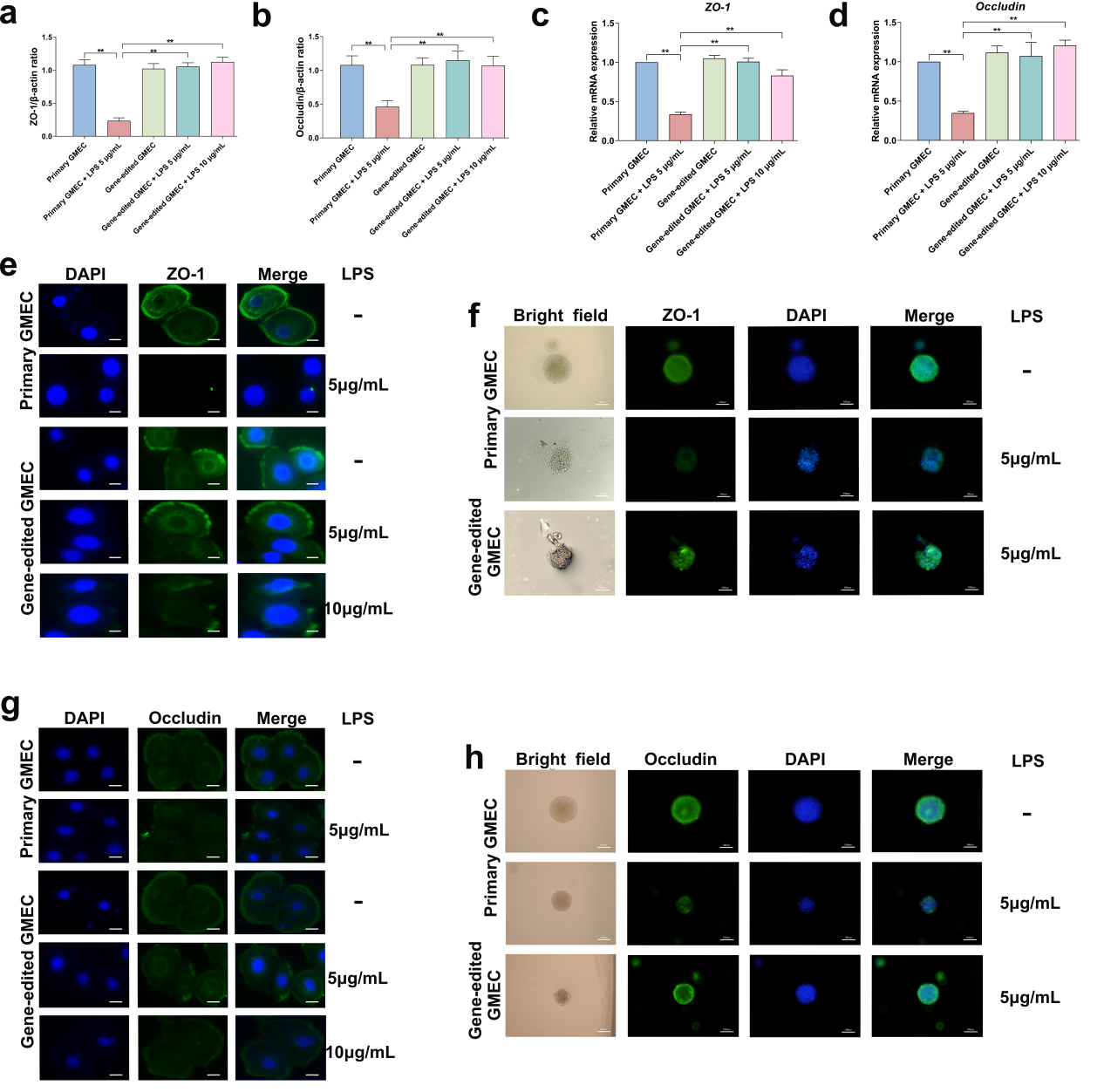
****Figure S16. Gene-edited GMEC alleviates TJ damage by high expression of LYZ under inflammatory conditions.** (a-b) The protein ratio of ZO-1, Occludin and β-actin. (c-d) Analysis of *ZO-1* and *Occludin* mRNA expression. (e) The protein expression of ZO-1 at the cell membrane was detected by IF. Scale bar: 10 µm. (f) The protein expression of ZO-1 in GMEC organoids was detected by IF. Scale bar: 100 µm. (g) The protein expression of Occludin at the cell membrane was detected by IF. Scale bar: 10 µm. (h) The protein expression of Occludin in GMEC organoids was detected by IF. Scale bar: 100 µm. Values are expressed as mean ± SEM (n=3 per group) by one-way ANOVA. *: indicates significant difference (*P* < 0.05), **: indicates that the difference is highly significant (*P* < 0.01) and ns: indicates no significant difference (*P* > 0.05).


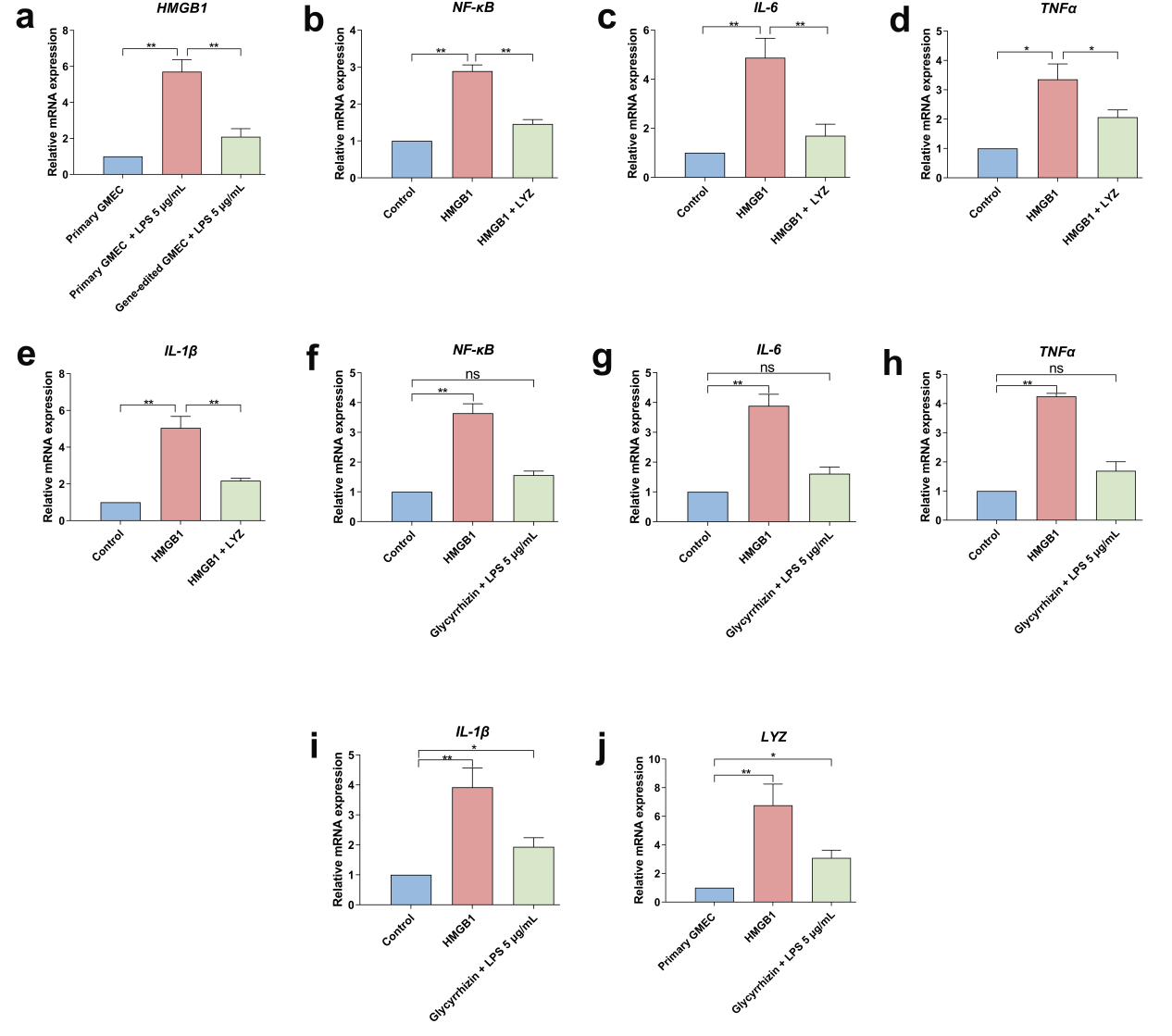
**Figure S17. *HMGB1*, *NF-κB*, *IL-6*, *IL-1β*, *TNFα* and *LYZ* mRNA expression analysis.** (a) Analysis of *HMGB1* mRNA expression. (b-e) Analysis of *NF-κB*, *IL-6*, *TNFα* and *IL-1β* mRNA expression. (f-j) Analysis of *NF-κB*, *IL-6*, *TNFα*, *IL-1β*, and *LYZ* mRNA expression. Values are expressed as mean ± SEM (n=3 per group) by one-way ANOVA. *: indicates significant difference (*P* < 0.05), **: indicates that the difference is highly significant (*P* < 0.01) and ns: indicates no significant difference (*P* > 0.05).


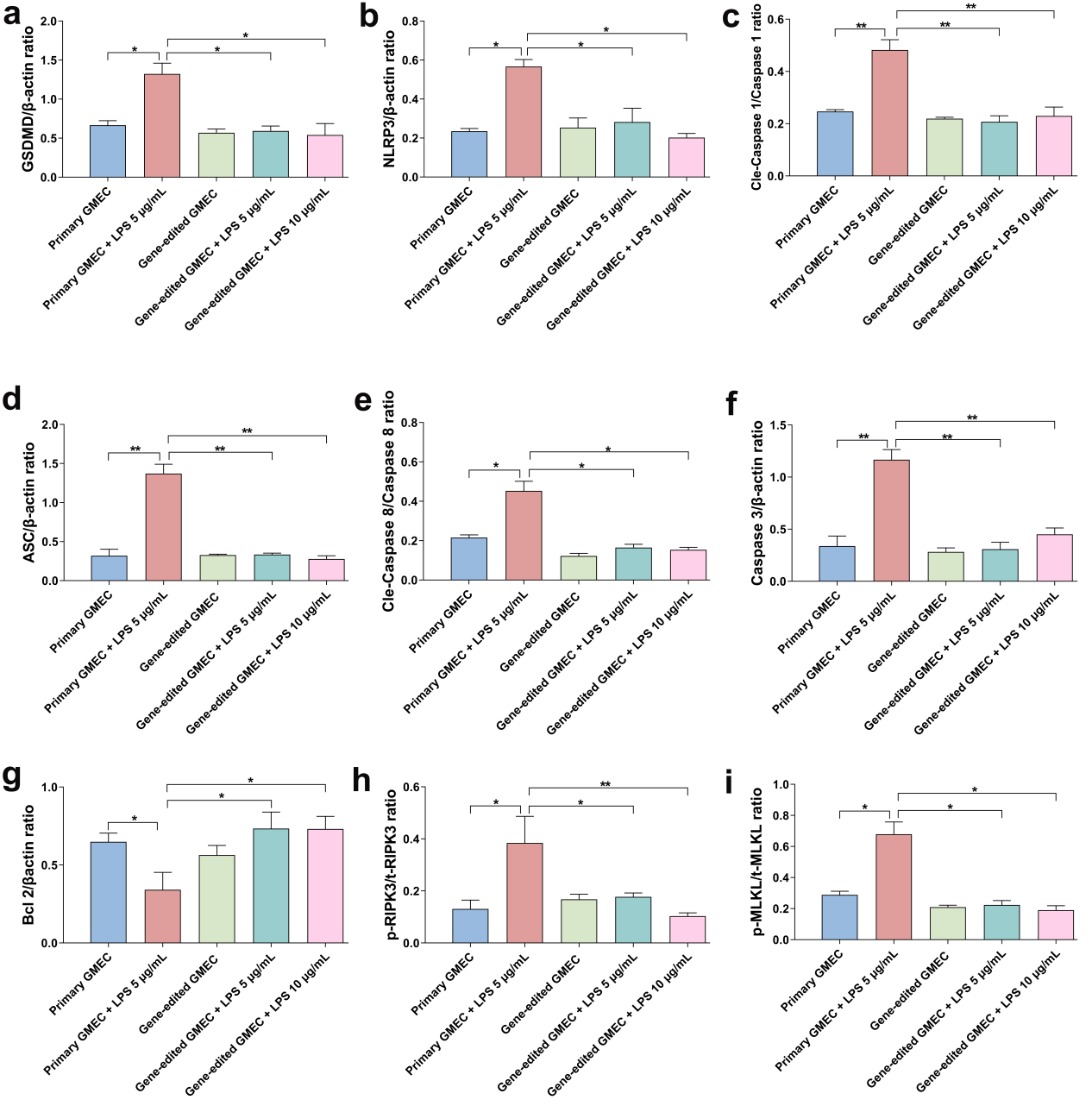
**Figure S18. Analysis of PANoptosis-related protein expression in GED goats mammary epithelial cells under inflammatory conditions.** (a-b) The protein ratio of GSDMD, NLRP3 and β-actin. (c) The protein ratio of Cle-Caspase 1 and Caspase 1. (d) The protein ratio of ASC and β-actin. (e) The protein ratio of Cle-Caspase 8 and Caspase 8. (f) The protein ratio of Caspase, Bcl2 and β-actin. (h) The protein ratio of p-RIPK3 and RIPK3. (i) The protein ratio of p-MLKL and MLKL. Values are expressed as mean ± SEM (n=3 per group) by one-way ANOVA. *: indicates significant difference (*P* < 0.05), **: indicates that the difference is highly significant (*P* < 0.01) and ns: indicates no significant difference (*P* > 0.05).

**
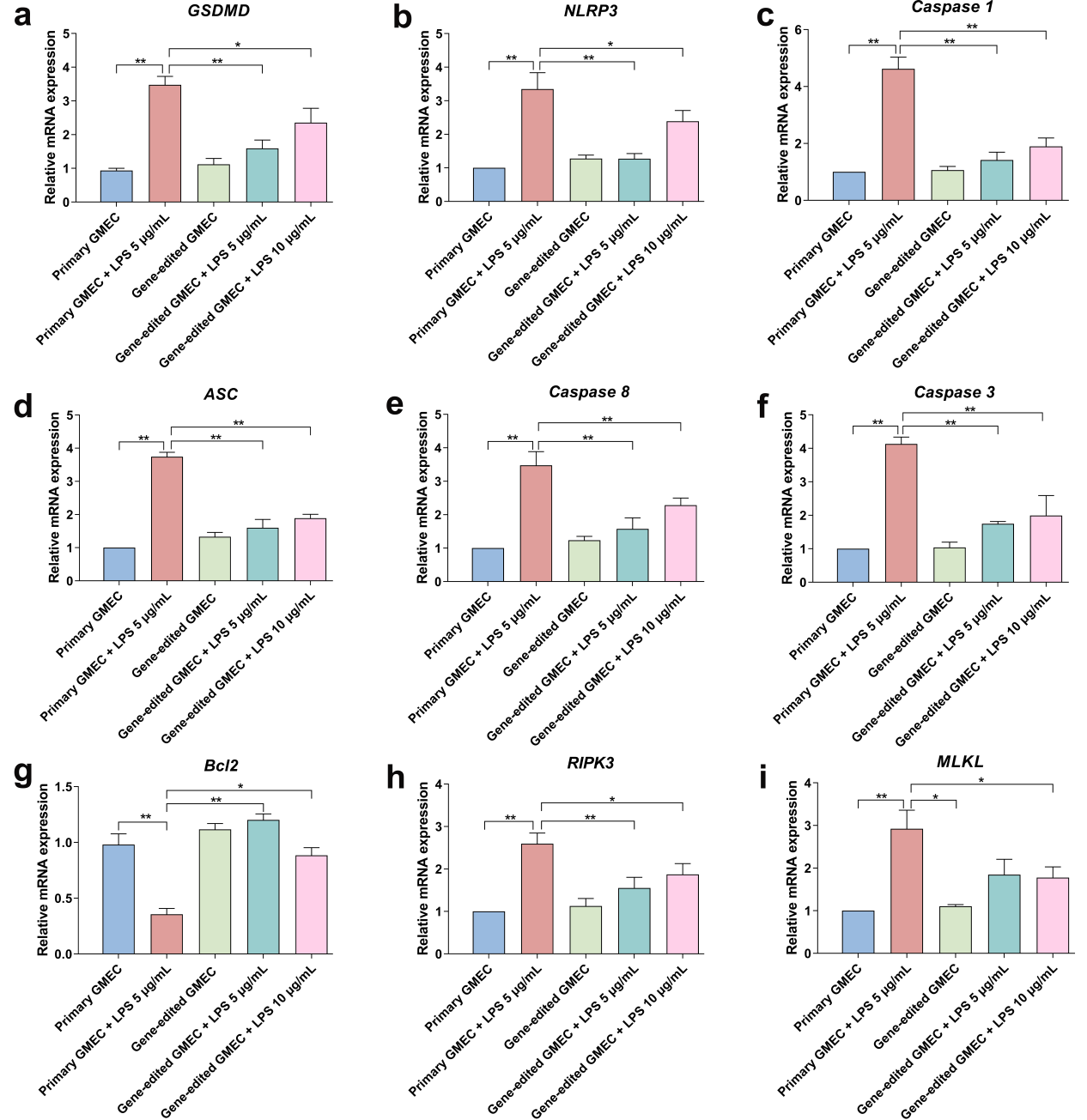
Figure S19. Analysis of mRNA expression of PANoptosis-related genes in GED goats mammary epithelial cells under inflammatory conditions.** (a-i) Analysis of mRNA expression of *GSDMD*, *NLRP3*, *Caspase 1*, *ASC*, *Caspase 8*, *Caspase 3*, *Bcl2*, *RIPK3* and *MLKL*. Values are expressed as mean ± SEM (n=3 per group) by one-way ANOVA. *: indicates significant difference (*P* < 0.05), **: indicates that the difference is highly significant (*P* < 0.01) and ns: indicates no significant difference (*P* > 0.05).

**
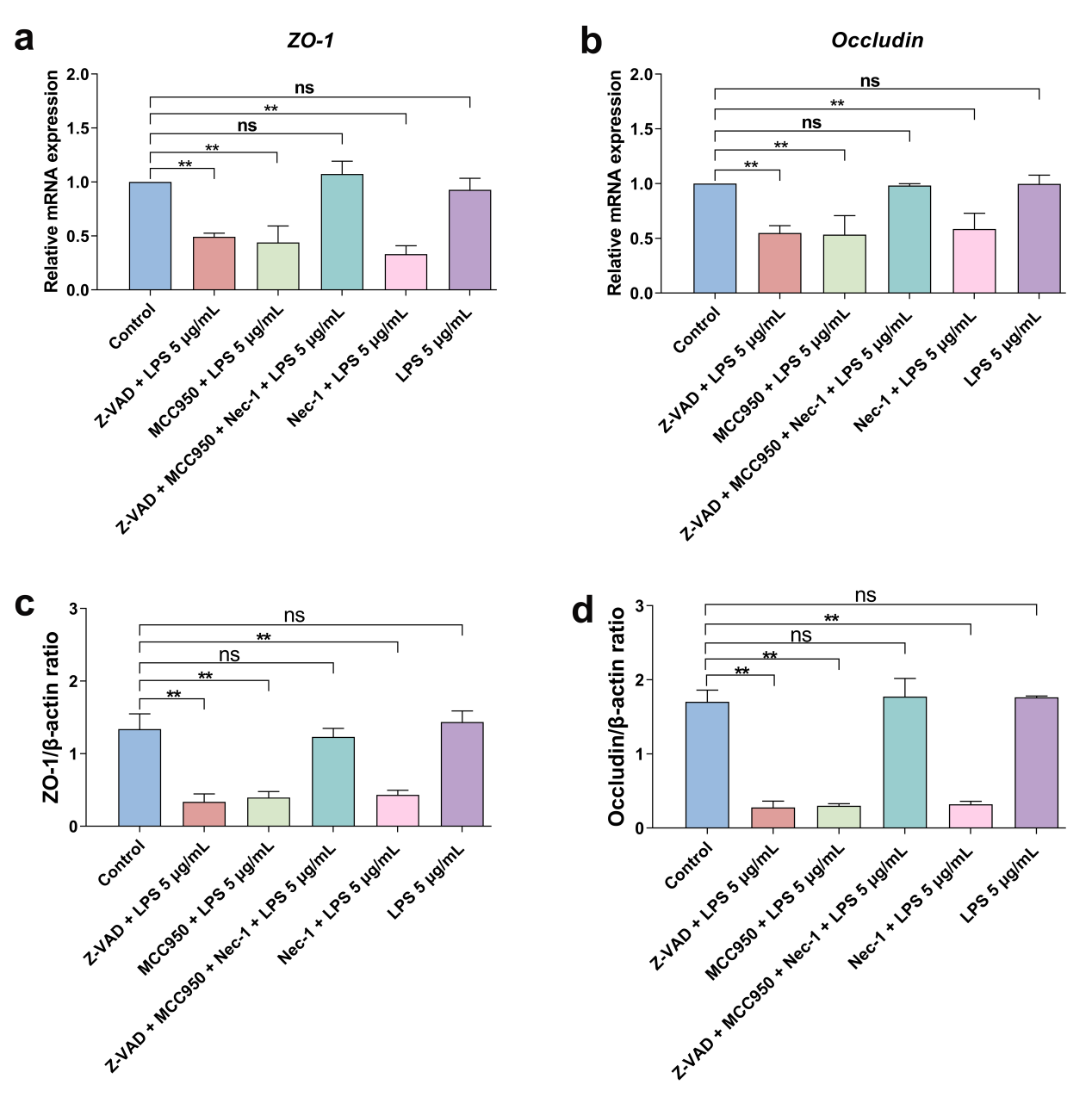
Figure S20. Inhibiting the activation of PANoptosis prevents downregulation of ZO-1 and Occludin mRNA and protein expression.** (a-b) Analysis of mRNA expression of *ZO-1* and *Occludin* after treatment with Z-VAD, MCC950 and Nec-1. (c-d) The protein ratio of ZO-1, Occludin and β-actin. Values are expressed as mean ± SEM (n=3 per group) by one-way ANOVA. *: indicates significant difference (*P* < 0.05), **: indicates that the difference is highly significant (*P* < 0.01) and ns: indicates no significant difference (*P* > 0.05).

| **Table S1 Primers for RT-PCR, gene full length cloning, and vector construction** | | | |
| --- | --- | --- | --- |
| **Gene** | | | **Primer sequence** |
| TLR4 TSS 1bp ~ -2000bp F | | | AACCCAGCACTGCTTTGAATAGGGGCAGTGCCCTGG |
| TLR4 TSS 1bp ~ -2000bp R | | | AAATGAGGGTAATAATATGTACCTCAAAGTATTCTT |
| TLR4 TSS 1bp ~ -1500bp F | | | AACCCAGCACTGCTTTGAATAGGGGCAGTGCCCTGG |
| TLR4 TSS 1bp ~ -1500bp R | | | ACGGCTACCGAGGAGCCCCGGCTCACCACACCTAGAG |
| TLR4 TSS 1bp ~ -1000bp F | | | AACCCAGCACTGCTTTGAATAGGGGCAGTGCCCTGG |
| TLR4 TSS 1bp ~ -1000bp R | | | CATCAATTCTTTGGCACTCAGCTTTCTTTATAGTCC |
| TLR4 TSS 1bp ~ -500bp F | | | AACCCAGCACTGCTTTGAATAGGGGCAGTGCCCTGG |
| TLR4 TSS 1bp ~ -500bp R | | | TTGAAGATGGCTGTCTTCTCCCTGCGCCTTCACGTC |
| LYZ TSS 1bp ~ -2000bp F | | | GGGGGAGAACAGAGAACTTTTCTTGGATCTTCTTC |
| LYZ TSS 1bp ~ -2000bp R | | | TGTTGACTGAGAAGCCAGACCTCCAGGCTGACCAG |
| RvP3 F | | | CTAGCAAAATAGGCTGTCCC |
| SSA-gRNA1 R | | | CTCGAATTCCTTGATGTAAGTGAAAGAGGAGAGTGCTCACATAGGACCTCTCACACACAG |
| SSA-gRNA2 R | | | CTCGAATTCCTAAAAGGCAGAGGAACCAGAGATCAACTCACATAGGACCTCTCACACACAG |
| SSA-gRNA3 R | | | CTCGAATTCCTTTGCCAACATCCGATGGATCATCAACTCACATAGGACCTCTCACACACAG |
| SSA-gRNA4 R | | | CTCGAATTCCTTTGATGCTTTTGAACTGTGGTGTTGCTCACATAGGACCTCTCACACACAG |
| SSA-gRNA5 R | | | CTCGAATTCCTTGTAAATTCTTAATTTATAAATCAACTCACATAGGACCTCTCACACACAG |
| SSA-gRNA6 R | | | CTCGAATTCCTTGGAAGAAGCACAAGCTGGAATCAACTCACATAGGACCTCTCACACACAG |
| LucRep F | | | CGAAGGTTGTGGATCTGGATACC |
| LucRep R | | | TAGCTGATGTAGTCTCAGTGAGC |
| Left homologous arm F | | | TGTCTAGGACTTGGACTTACAGATCATCTTAACC |
| Left homologous arm R | | | AGAAGACTCTTGAGAGTCCCTTGGACTGCAAGGA |
| Right homologous arm F | | | TGTTGGCAATTTGATCTCTGGTTCCTCTGCCTTT |
| Right homologous arm R | | | CTGAAGAATTGATGCTTTTGAACTGTGGTGTTGG |
| IRS F | | | GGACTATAAAGAAAGCTGAGTGCCAAAGAATTG |
| IRS R | ATCTTCTCTCTGCGTGTGCTGGGTCTTAATTTCCTT | | |
| 5' junction F | AGAAAACAAAACAAATGGAGATTCTCATCTTATG | | |
| 5' junction R | GTAGCCTTCGGGCATGGCGGACTTGAAGAAGT | | |
| 3' junction F | CTTAGGATGTCAGAATGTCCCTCTTCAAACATAA | | |
| 3' junction R | GCGCGGTCCTTCGGGCACCTCGACGTCGGCGG | | |
| 5'arm F | CTGCATTGCCTTCAGCTCAAAATTTTGTCAAAGG | | |
| 3'arm R | AGAAATGCTGGGCTGGAAGAAGCACAAGCTGGAA | | |
| TLR4([NM_001285574](https://www.ncbi.nlm.nih.gov/nuccore/NM_001285574).1) F | | | TTCAACCGTATCACGGCCTC |
| TLR4([NM_001285574](https://www.ncbi.nlm.nih.gov/nuccore/NM_001285574).1) R | | TGACCCACTGCAGGAAACTC | |
| MyD88(XM_013973392.2) F | | CCCCATCAAGTACAAGCCAATG | |
| MyD88(XM_013973392.2) R | | CGAGGCGAGTCCAGAACCAG | |
| IKBα(XM_018066509.1) F | | | GCTCACCGAGGACGGAGACT |
| IKBα(XM_018066509.1) R | | | TGCCCAGGTAGCCATGAATAG |
| NF-κB(XM_018043384.1) F | | | TGGCGAGAGGAGCACAGACAC |
| NF-κB(XM_018043384.1) R | | | TGACCAGCGAGATGCGGACTG |
| IL-6(NM_001285640.1) F | | | TGATGACTTCTGCTTTCCCTAC |
| IL-6(NM_001285640.1) R | | | AACCTTTGCGTTCTTTACCC |
| IL-1β(XM_013967700.2) F | | | CATGTGTGCTGAAGGCTCTC |
| IL-1β(XM_013967700.2) R | | | AGTGTCGGCGTATCACCTTT |
| TNFα(XM_005696606) F | | | CAAGTAACAAGCCGGTAGCC |
| TNFα(XM_005696606) R | | | AGATGAGGTAAAGCCCGTCA |
| LYZ(NM_001285711.1) F | | | GATTTGGAATGGATGGCTTTA |
| LYZ(NM_001285711.1) R | | | AACCCTGAATGTAACTGGTGAG |
| GSDMD(XM_018058675.1) F | | | CTGGTTATTGGCTCTGACTGGG |
| GSDMD(XM_018058675.1) R | | | CCTCATGGAGGCAAAGCTGAA |
| NLRP3(XM_005682796.3) F | | | CCATAGGCAAGATTGTAAGC |
| NLRP3(XM_005682796.3) R | | | GTGTTTCTAGGGCACGTTTT |
| Caspase1(XM_018058933.1) F | | | TGCTACTAGAAGGTCTGGGATA |
| Caspase1(XM_018058933.1) R | | | GACTGAGGCAATTACGGTTG |
| ASC(XM_005697733.3) F | | | AGTTACTATCTGGAGGCATACGGT |
| ASC(XM_005697733.3) R | | | AGTCCTGGCTTGGCTATCTTCT |
| Caspase8(XM_018060934.1) F | | | CCTGGCTGCCCTCAAGTTCC |
| Caspase8(XM_018060934.1) R | | | TGCTCCCGTGCTATGCTAAA |
| Bcl2(XM_018039337) F | | | CGCATCGTGGCCTTCTTTGA |
| Bcl2(XM_018039337) R | | | TCCTCCAGCCTCCGTTGTCC |
| Caspase3(XM_018041755) F | | | CCTGGACTGTGGTATTGAGA |
| Caspase3(XM_018041755) R | TAACCCGAGTAAGAATGTGC | | |
| RIPK3(XM_018054645.1) F | GCGGATTTGGCTCTGTCTTC | | |
| RIPK3(XM_018054645.1) R | GGTTCTTTCGGTCGTTCTTTT | | |
| MLKL(XM_013970972.2) F | GGTCAGATTCACAGAAACCCAA | | |
| MLKL(XM_013970972.2) R | TAGGCACGGCAATCATCAAC | | |
| ZO-1(XM_018066118.1) F | ACAGATGCAAAGACGCTGAT | | |
| ZO-1(XM_018066118.1) R | CTTGTGGTGAGTAGGGAGGAT | | |
| Occlduin(XM_018065677.1) F | CCAGCGTTGTAAGGTCAGGC | | |
| Occlduin(XM_018065677.1) R | TTTCCGTCGGTCGTAATCTC | | |
| LGR5(XM_005679712.3) F | AACCTGCGAAGCCTTCAGTC | | |
| LGR5(XM_005679712.3) R | CTGTGGAGCCCATCAAAGCA | | |
| Sox2(NM_001285672.1) F | TGCACAACTCGGAAATCAGCAA | | |
| Sox2(NM_001285672.1) R | GTGGGAAGAAGAGGTAACCACAGG | | |
| Sox9(XM_018063905.1) F | TCGCCCAACGCCATCTTCAA | | |
| Sox9(XM_018063905.1) R | GTACTGGTCGAACTCGTGGAC | | |
| HMGB1([XM_018056617.1](https://www.ncbi.nlm.nih.gov/nuccore/XM_018056617.1)) F | AATAACACTGCTGCGGATGA | | |
| HMGB1([XM_018056617.1](https://www.ncbi.nlm.nih.gov/nuccore/XM_018056617.1)) R | AGCCTTGACAACTCCCTTTT | | |
| GAPDH(XM_005680968.3) F | TGGCAAGTTCCACGGCACAG | | |
| GAPDH(XM_005680968.3) R | TTCTCCAGGCGGCAGGTCAG | | |

**Table S2 Sequence information for ISDra2 TnpB and reRNA**

| **Sequence name** | **Sequence information** |
| --- | --- |
| ISDra2 TnpB Sequence | ATGATAAGGAATAAGGCTTTCGTGGTCAGGCTGTACCCAAATGCGGCTCAGACTGAACTGATTAACCGCACGCTGGGTAGCGCAAGGTTCGTCTACAACCACTTCCTTGCCCGTCGCATTGCGGCCTACAAGGAAAGCGGGAAGGGACTGACCTACGGGCAAACGAGTAGCGAACTGACCCTTCTGAAGCAGGCTGAAGAAACCTCCTGGCTCTCGGAAGTAGATAAGTTTGCTTTGCAGAACTCGCTGAAAAACCTTGAGACCGCGTACAAGAACTTCTTTCGGACTGTGAAGCAGTCCGGTAAAAAGGTAGGATTCCCACGTTTCAGAAAGAAGCGCACGGGAGAGTCCTACCGGACTCAATTCACCAACAACAACATCCAAATTGGGGAAGGTAGGCTCAAACTTCCTAAGCTGGGATGGGTGAAAACCAAGGGCCAGCAGGATATTCAAGGGAAGATTCTGAATGTCACTGTGCGCCGTATTCACGAAGGCCATTACGAAGCGTCCGTTCTCTGTGAAGTCGAGATTCCCTACCTGCCTGCGGCTCCCAAGTTTGCAGCGGGTGTGGATGTCGGCATCAAGGATTTTGCCATCGTGACCGATGGCGTGAGGTTTAAGCATGAACAGAATCCGAAATATTACCGCTCCACCCTGAAAAGACTTCGTAAAGCTCAGCAAACCCTGTCCAGACGGAAGAAGGGCAGCGCACGTTACGGGAAAGCGAAAACCAAGCTGGCTCGGATTCACAAGCGCATTGTCAATAAGCGTCAGGATTTCCTTCACAAGCTCACCACCTCCCTGGTGCGTGAGTACGAAATCATCGGAACCGAACACCTTAAACCCGACAACATGCGGAAAAATCGCCGCCTTGCACTGAGCATCAGTGATGCGGGCTGGGGTGAGTTCATCCGGCAGTTGGAATACAAGGCAGCGTGGTACGGGCGACTGGTATCTAAAGTCAGCCCCTACTTTCCATCTAGCCAGTTGTGTCATGACTGCGGATTCAAGAATCCCGAAGTGAAGAATCTTGCCGTCCGTACATGGACTTGCCCGAACTGTGGGGAAACCCATGACCGAGACGAGAACGCTGCGCTGAACATTCGGCGTGAAGCGTTGGTGGCTGCGGGAATCTCAGACACCTTAAACGCTCATGGAGGCTATGTCAGACCTGCTTCGGCGGGCAATGGTCTGCGAAGTGAGAATCACGCGACTTTAGTCGTG |
| TnpB amino acid sequence | MIRNKAFVVRLYPNAAQTELINRTLGSARFVYNHFLARRIAAYKESGKGLTYGQTSSELTLLKQAEETSWLSEVDKFALQNSLKNLETAYKNFFRTVKQSGKKVGFPRFRKKRTGESYRTQFTNNNIQIGEGRLKLPKLGWVKTKGQQDIQGKILNVTVRRIHEGHYEASVLCEVEIPYLPAAPKFAAGVDVGIKDFAIVTDGVRFKHEQNPKYYRSTLKRLRKAQQTLSRRKKGSARYGKAKTKLARIHKRIVNKRQDFLHKLTTSLVREYEIIGTEHLKPDNMRKNRRLALSISDAGWGEFIRQLEYKAAWYGRLVSKVSPYFPSSQLCHDCGFKNPEVKNLAVRTWTCPNCGETHDRDENAALNIRREALVAAGISDTLNAHGGYVRPASAGNGLRSENHATLVV |
| reRNA (sgRNA1) | GATTCAAGAATCCCGAAGTGAAGAATCTTGCCGTCCGTACATGGACTTGCCCGAACTGTGGGGAAACCCATGACCGAGACGAGAACGCTGCGCTGAACATTCGGCGTGAAGCGTTGGTGGCTGCGGGAATCTCAGACACCTTAAACGCTCATGGAGGCTATGTCAGACCTGCTTCGGCGGGCAATGGTCTGCGAAGTGAGAATCACGCGACTTTAGTCGTGTGAGGTTCAACACTCTCCTCTTTCACTTAC |
| reRNA (sgRNA2) | GATTCAAGAATCCCGAAGTGAAGAATCTTGCCGTCCGTACATGGACTTGCCCGAACTGTGGGGAAACCCATGACCGAGACGAGAACGCTGCGCTGAACATTCGGCGTGAAGCGTTGGTGGCTGCGGGAATCTCAGACACCTTAAACGCTCATGGAGGCTATGTCAGACCTGCTTCGGCGGGCAATGGTCTGCGAAGTGAGAATCACGCGACTTTAGTCGTGTGAGGTTCAACTCTGGTTCCTCTGCCTTTT |
| reRNA (sgRNA3) | GATTCAAGAATCCCGAAGTGAAGAATCTTGCCGTCCGTACATGGACTTGCCCGAACTGTGGGGAAACCCATGACCGAGACGAGAACGCTGCGCTGAACATTCGGCGTGAAGCGTTGGTGGCTGCGGGAATCTCAGACACCTTAAACGCTCATGGAGGCTATGTCAGACCTGCTTCGGCGGGCAATGGTCTGCGAAGTGAGAATCACGCGACTTTAGTCGTGTGAGGTTCAAGATCCATCGGATGTTGGCAA |
| reRNA (sgRNA4) | GATTCAAGAATCCCGAAGTGAAGAATCTTGCCGTCCGTACATGGACTTGCCCGAACTGTGGGGAAACCCATGACCGAGACGAGAACGCTGCGCTGAACATTCGGCGTGAAGCGTTGGTGGCTGCGGGAATCTCAGACACCTTAAACGCTCATGGAGGCTATGTCAGACCTGCTTCGGCGGGCAATGGTCTGCGAAGTGAGAATCACGCGACTTTAGTCGTGTGAGGTTCAACAACACCACAGTTCAAAAGC |
| reRNA (sgRNA5) | GATTCAAGAATCCCGAAGTGAAGAATCTTGCCGTCCGTACATGGACTTGCCCGAACTGTGGGGAAACCCATGACCGAGACGAGAACGCTGCGCTGAACATTCGGCGTGAAGCGTTGGTGGCTGCGGGAATCTCAGACACCTTAAACGCTCATGGAGGCTATGTCAGACCTGCTTCGGCGGGCAATGGTCTGCGAAGTGAGAATCACGCGACTTTAGTCGTGTGAGGTTCAATTATAAATTAAGAATTTACA |
| reRNA (sgRNA6) | GATTCAAGAATCCCGAAGTGAAGAATCTTGCCGTCCGTACATGGACTTGCCCGAACTGTGGGGAAACCCATGACCGAGACGAGAACGCTGCGCTGAACATTCGGCGTGAAGCGTTGGTGGCTGCGGGAATCTCAGACACCTTAAACGCTCATGGAGGCTATGTCAGACCTGCTTCGGCGGGCAATGGTCTGCGAAGTGAGAATCACGCGACTTTAGTCGTGTGAGGTTCAATCCAGCTTGTGCTTCTTCCA |

**Table S3 The predicted potential off-target sites of TnpB**

| **Target gene** | **The potential off-target sequence** |
| --- | --- |
| Goat LYZ-OT1 | TTGATGaTTTcaAACTGTGGTaTcG |
| Goat LYZ-OT2 | TTGATGCTTTTGAAtTGTGtTtgaG |
| Goat LYZ-OT3 | TTGATGCTTTcaAAtTGTGtTGcTG |
| Goat LYZ-OT4 | TTGATGtTTTTGAACTaTGaTacTG |
| Goat LYZ-OT5 | TTGATGCcTTctAACTaTGGTGcTG |
| Goat LYZ-OT6 | TTGATGCTTTTGAgCTaTGtTGcTG |
| Goat LYZ-OT7 | TTGATGCcTTcaAACTGTGGTGcTG |

| **Table S4 Antibody information** | | | | | | |
| --- | --- | --- | --- | --- | --- | --- |
| **Protein name** | | **Company** | **Catalogue no** | | **Dilution rate** | **WB/IF** |
| TLR4 | | Proteintech | 19811-AP | | 1:500 | WB |
| Phospho-IκBα | | Proteintech | 82349-1-RR | | 1:500 | WB |
| IκBα | | Proteintech | 10268-1-AP | | 1:500 | WB |
| MyD88 | | Proteintech | 23230-1-AP | | 1:500 | WB |
| Phospho-NF-κB P65 | | Cell Signaling Technology | 3033 | | 1:500 | WB |
| NF-κB P65 | | Proteintech | 10745-1-AP | | 1:100 | WB |
| ZO-1 | | Proteintech | 21773-1-AP | | 1:500 | WB |
| Occludin | | Proteintech | 66378-1-Ig | | 1:500 | WB |
| HMGBI | | Proteintech | 10829-1-AP | | 1:500 | WB |
| GSDMD | | Proteintech | 20770-1-AP | | 1:500 | WB |
| NLRP3 | | Proteintech | 27458-1-AP | | 1:500 | WB |
| Caspase1 | | Proteintech | 22915-1-AP | | 1:500 | WB |
| ASC | | Proteintech | 10500-1-AP | | 1:500 | WB |
| Caspase 8 | | Proteintech | 13423-1-AP | | 1:500 | WB |
| BCl2 | | Proteintech | 68103-1-Ig | | 1:500 | WB |
| Caspase3 | | Proteintech | 19677-1-AP | | 1:500 | WB |
| p-RIPK3 | | Cell Signaling Technology | 91702 S | | 1:500 | WB |
| RIPK3 | | Proteintech | 17563-1-AP | | 1:500 | WB |
| p-MLKL | | Cell Signaling Technology | 37333 | | 1:500 | WB |
| MLKL | | Proteintech | 66675-1-Ig | | 1:500 | WB |
| IL-6 | | Proteintech | 66146-1-Ig | | 1:500 | WB |
| TNFα | | Proteintech | 60291-1-Ig | | 1:500 | WB |
| IL-1β | | Proteintech | 16806-1-AP | | 1:500 | WB |
| β-actin | | Proteintech | 20536-1-AP | | 1:500 | WB |
| HRP-conjugated Affinipure GoatAnti-Mouse IgG(H+L) | | Proteintech | SA00001-1 | | 1:5000 | WB |
| HRP-conjugated Affinipure Goat Anti-Rabbit IgG(H+L) | | Proteintech | SA00001-2 | | 1:5000 | WB |
| Phospho-NF-κB P65 | | Cell Signaling Technology | 3033 | | 1:100 | IF |
| ZO-1 | | Proteintech | 21773-1-AP | | 1:100 | IF |
| Occludin | | Proteintech | 66378-1-Ig | | 1:100 | IF |
| NLRP3 | | Proteintech | 27458-1-AP | | 1:100 | IF |
| CD49f | | Santa Cruz Biotechnology | SC-19622 | | 1:100 | IF |
| Alexa Fluor 488 | | Beyotime | A0428 | | 1:500 | IF |
| Alexa Fluor 488 | | Beyotime | A0423 | | 1:500 | IF |
| **Table S5 GMEC organoid culture medium** | | | | | | |
| **Drug** | **Dosage** | | | **Company** | | |
| FBS | 5%-10% | | | Gibco, USA | | |
| ITS | 1%-3% | | | Sigma, USA | | |
| B-27 | 2%-4% | | | Gibco, USA | | |
| EGF | 5 ng/mL | | | Novus, USA | | |
| b FGF | 5 ng/mL | | | Gibco, USA | | |
| Penicillin-streptomycin | 50 U/mL | | | Gibco, USA | | |
| L-Gln | 0.1 mmol/L | | | Sigma, USA | | |
| Noggin | 10 ng/mL | | | Novoprotein, China | | |
| Rspondin-1 | 10 ng/mL | | | Novoprotein, China | | |
| E2 | 100 nM | | | Sigma, USA | | |
| Wnt-3a | 10 ng/mL | | | Sigma, USA | | |
| Y-27632 | 5 μM | | | MCE, USA | | |
| SB202190 | 5 μM | | | MCE, USA | | |
| A-8301 | 1 μM | | | MCE, USA | | |
